# Plasticity of the *MFS1* Promoter Leads to Multidrug Resistance in the Wheat Pathogen *Zymoseptoria tritici*

**DOI:** 10.1128/mSphere.00393-17

**Published:** 2017-10-25

**Authors:** Selim Omrane, Colette Audéon, Amandine Ignace, Clémentine Duplaix, Lamia Aouini, Gert Kema, Anne-Sophie Walker, Sabine Fillinger

**Affiliations:** a UMR BIOGER, INRA, AgroParisTech, Université Paris-Saclay, Thiverval-Grignon, France; b Wageningen University, Plant Research International, Wageningen, The Netherlands; Carnegie Mellon University

**Keywords:** antifungal resistance, bulk progeny analysis, efflux pumps, multidrug resistance, repeated elements, transcriptional regulation

## Abstract

The ascomycete *Zymoseptoria tritici* is the causal agent of Septoria leaf blotch on wheat. Disease control relies mainly on resistant wheat cultivars and on fungicide applications. The fungus displays a high potential to circumvent both methods. Resistance against all unisite fungicides has been observed over decades. A different type of resistance has emerged among wild populations with multidrug-resistant (MDR) strains. Active fungicide efflux through overexpression of the major facilitator gene *MFS1* explains this emerging resistance mechanism. Applying a bulk-progeny sequencing approach, we identified in this study a 519-bp long terminal repeat (LTR) insert in the *MFS1* promoter, a relic of a retrotransposon cosegregating with the MDR phenotype. Through gene replacement, we show the insert as a mutation responsible for *MFS1* overexpression and the MDR phenotype. Besides this type I insert, we found two different types of promoter inserts in more recent MDR strains. Type I and type II inserts harbor potential transcription factor binding sites, but not the type III insert. Interestingly, all three inserts correspond to repeated elements present at different genomic locations in either IPO323 or other *Z. tritici* strains. These results underline the plasticity of repeated elements leading to fungicide resistance in *Z. tritici* and which contribute to its adaptive potential.

**IMPORTANCE** Disease control through fungicides remains an important means to protect crops from fungal diseases and to secure the harvest. Plant-pathogenic fungi, especially *Zymoseptoria tritici*, have developed resistance against most currently used active ingredients, reducing or abolishing their efficacy. While target site modification is the most common resistance mechanism against single modes of action, active efflux of multiple drugs is an emerging phenomenon in fungal populations reducing additionally fungicides’ efficacy in multidrug-resistant strains. We have investigated the mutations responsible for increased drug efflux in *Z. tritici* field strains. Our study reveals that three different insertions of repeated elements in the same promoter lead to multidrug resistance in *Z. tritici*. The target gene encodes the membrane transporter MFS1 responsible for drug efflux, with the promoter inserts inducing its overexpression. These results underline the plasticity of repeated elements leading to fungicide resistance in *Z. tritici*.

## Abstract (precis)

Disease control through fungicides remains an important means to protect crops from fungal diseases and to secure the harvest. Plant-pathogenic fungi, especially *Zymoseptoria tritici*, have developed resistance against most currently used active ingredients, reducing or abolishing their efficacy. While target site modification is the most common resistance mechanism against single modes of action, active efflux of multiple drugs is an emerging phenomenon in fungal populations reducing additionally fungicides’ efficacy in multidrug-resistant strains. We have investigated the mutations responsible for increased drug efflux in *Z. tritici* field strains. Our study reveals that three different insertions of repeated elements in the same promoter lead to multidrug resistance in *Z. tritici*. The target gene encodes the membrane transporter MFS1 responsible for drug efflux, with the promoter inserts inducing its overexpression. These results underline the plasticity of repeated elements leading to fungicide resistance in *Z. tritici*.

## INTRODUCTION

Wheat is the most widely grown crop in the world. It is subject to several diseases, principally due to fungal pests. Its major disease in Europe and North America is Septoria leaf blotch (SLB), caused by *Zymoseptoria tritici* (formerly *Mycosphaerella graminicola*) (1, 2). The disease pressure of SLB depends on epidemical and environmental factors (3) and can be reduced by adapted agronomical practices (e.g., crop rotation) and intelligent use of less-susceptible varieties (4). Finally, SLB prevention strongly relies on the application of fungicides, namely, inhibitors of sterol demethylation (DMIs [including azoles]), inhibitors of mitochondrial complex II (SDHIs), and the multisite inhibitor chlorothalonil. According to disease pressure, spray programs targeting SLB range from one (southern Europe) to four (Ireland and United Kingdom) sprays and around two treatments per year in France. The first treatment generally includes mixtures of azoles and chlorothalonil. The second spray aims to protect the key stage when the first leaf is emerging. Mixtures of azoles and SDHIs are often applied.

*Z. tritici* populations have developed resistance to all unisite fungicides, but to different extents. Azole resistance is generalized in Europe since the 1990s and now affects the field efficacy of the molecules, but SDHI resistance has just emerged and does not yet affect the efficacy of this mode of action (5). Resistance is principally due to target site modification or overexpression (6–8).

Multidrug resistance (MDR) operating through increased drug efflux is a resistance mechanism recently detected in some field isolates of *Z. tritici*. Since it is associated with azole target site resistance, it confers high resistance factors to this class of inhibitors, whereas only low resistance levels toward SDHIs are recorded (8).

The phenomenon of MDR is well known from human cancer cells and antibiotic-resistant bacteria (9, 10). In fungi, *Saccharomyces cerevisiae* has served as a model organism to elucidate MDR (also termed PDR for “pleiotropic drug resistance”) and its regulation. It has also been extensively studied in several pathogenic yeast species (e.g., *Candida albicans* and *Candida glabrata*). For reviews, we refer the reader to some excellent papers (11–15). Globally, MDR is conferred by constitutive overexpression of membrane transporter genes either of the ATP-binding cassette (ABC) type or of the major facilitator superfamily (MFS). These transporters expel drugs outside the cell, thereby reducing the intracellular drug concentrations. Their specificity can be more or less broad (16). Constitutive overexpression of membrane transporters in clinical isolates of *C. albicans* was found to be due to gain-of-function mutations in the transcription factors Tac1 or Mrr1, controlling, respectively, the expression of the ABC transporter gene *CDR1* or of the MFS protein-encoding *MDR1* gene.

In the phytopathogenic fungi *Botrytis cinerea*, *Sclerotinia homeocarpa*, and *Oliculimacula yallundae*, MDR has also been described for field isolates (17–21). The mutations responsible for MDR in *B. cinerea* field strains have been identified. They correspond either to a retroelement-like insert in the promoter of the *B. cinerea mfsM2* (*BcmfsM2*) gene or to gain-of-function mutations in the transcription factor Mrr1 controlling the expression of the ABC transporter gene *BcatrB* (22, 23). Strains harboring either or both mutations are frequent among wild *B. cinerea* populations (23–25).

In a recent study, we have shown that fungicide efflux was at work in two *Z. tritici* MDR field isolates (26). Both strains, as well as other MDR strains tested, constitutively overexpress the gene *MFS1* (originally named *MgMFS1* for *Mycosphaerella graminicola MFS1*) encoding an MFS transporter capable of transporting a wide variety of molecules (27). Its inactivation in one MDR strain abolished the MDR phenotype, revealing that the MFS1 protein is necessary for the MDR phenotype at least in this strain. In both analyzed MDR strains, a 519-bp insert was detected in the *MFS1* promoter, a putative relic of an ancient long terminal repeat (LTR) retrotransposon. Other, but not all, field MDR strains proved to have this promoter insert as well, suggesting a potential role in MDR (26). In this study, we address the question of the mutations responsible for the MDR phenotype in the previously characterized MDR strains.

We used bulk segregant analysis (BSA) in order to map mutations responsible for MDR in *Z. tritici*. BSA is a genotyping method adapted to monogenic traits (28). It is based on the establishment of two phenotypically dissimilar pools derived from an offspring population. These pools are genotyped, and markers linked to the phenotype according to their allelic frequencies are selected to determine the locus of interest. With the rise of next-generation sequencing (NGS), statistical tools have been developed for BSA phenotyping to uncover quantitative trait loci (QTL) (29, 30) and others (31). BSA has been applied to identify genomic loci contributing to natural polymorphism (32) or mutant phenotypes (33). In fungi, the technique of BSA was successfully used to identify mutations responsible for cell cycle and developmental processes, respectively, in *Neurospora crassa* (34) and *Sordaria macrospora* (35). Due to its haploid characterized and annotated genome (36) and the possibility of performing sexual crosses between different strains (37), *Z. tritici* is suitable for BSA to map the mutation or mutations responsible for the MDR phenotypes.

In this work, we mapped the mutation responsible for the MDR phenotype in the two previously characterized field strains using BSA. After functional validation of the responsible mutation, the 519-bp promoter insert of the *MFS1* gene, we screened *Z. tritici* field strains for the *MFS1* promoter genotype. Interestingly two other inserts were identified in the same promoter only in *Z. tritici* MDR field strains overexpressing *MFS1*.

## RESULTS

### Genetic mapping of *mdr loci* in two MDR field strains.

To check whether MDR phenotypes described in reference 8 are driven by allelic mutations, we performed a cross between strains 09-ASA-3apz and 09-CB1. Rapid discrimination of MDR strains from sensitive ones consists of a growth test using fungicides that are not used in agriculture, such as tolnaftate and terbinafine (18), both squalene epoxidase inhibitors typically used against human fungal infections (38, 39). The progeny of 140 strains were analyzed on tolnaftate. Growth tests did not reveal any sensitive isolate (Table 1), suggesting that the two parental *mdr* mutations are closely linked on the same chromosome, in a common genomic region.

**TABLE 1:** Crosses used in this study and progeny segregation by tolnaftate sensitivity or resistance

Cross	Strains crossed	No. of strains^a^:		
		Sensitive	MDR	Total
1	09-ASA-3apz (MDR) × 09-CB1 (MDR)	0	140	140
2	09-ASA-3apz (MDR) × IPO94269 (sensitive)	149	148	297
3	09-CB1 (MDR) × IPO323 (sensitive)	111	97	208

a Shown is the progeny segregation according to sensitivity or resistance (MDR) to 2 µg ml^−1^ tolnaftate.

Both MDR strains were then crossed to the sequenced sensitive strains IPO94269 and IPO323, respectively. These two crosses were essential to map the *mdr loci*. We isolated and determined the phenotypes of 297 and 208 progeny strains, respectively. Crosses 2 and 3 generated, respectively, 50% and 47% MDR progeny strains (Table 1). In both cases, the ratio of MDR versus sensitive strains was in agreement with a single mutation responsible for the MDR phenotype. However, we need to underline that the selection of progeny strains was not completely unbiased after the elimination of mixtures among progeny strains. Therefore, statistical tests cannot be applied to offspring segregation.

**FIG 1:**
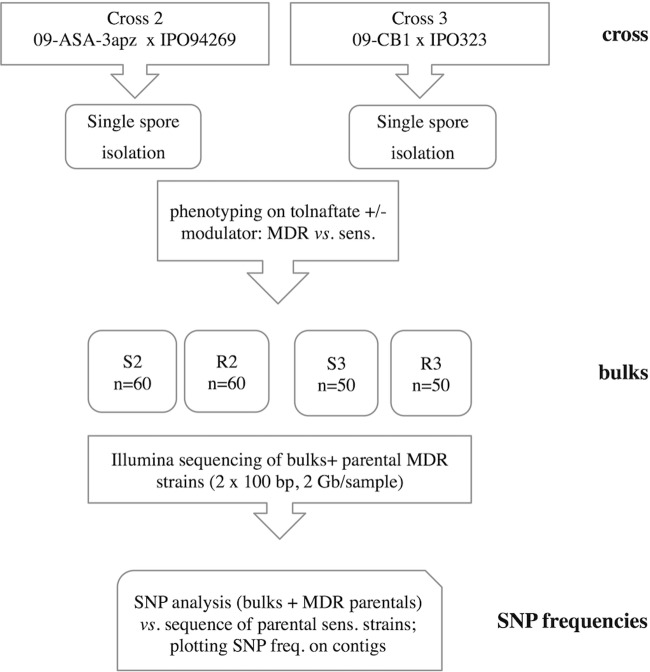
Flowchart of the applied BSA procedure. The bulks of progeny strains are designated sensitive (sens. [S]) or resistant (R) to tolnaftate, and the numbers 2 and 3 refer to the crosses listed in Table 1.

To map both *mdr* mutations, we decided to perform a bulk progeny sequencing approach (BSA) as developed for different phenotypes in other fungal species (34, 35). MDR and sensitive progeny strains were selected on the basis of maximal phenotypic dissimilarities by growth tests in medium supplemented with tolnaftate and the membrane transporter inhibitor verapamil to constitute resistant (R) or sensitive (S) bulks. We adjusted the number of progeny strains per bulk to *n* = 50 (cross 3: 09-CB1 × IPO323) and *n* = 60 (cross 2: 09-ASA-3apz × IPO94629). In order to map the *mdr* mutations, we adopted a 3-step protocol (Fig. 1) involving (i) DNA extraction from each of the four bulks, (ii) Illumina DNA sequencing, and (iii) sequence mapping to the reference sequence of the parental sensitive strain.

**FIG 2:**
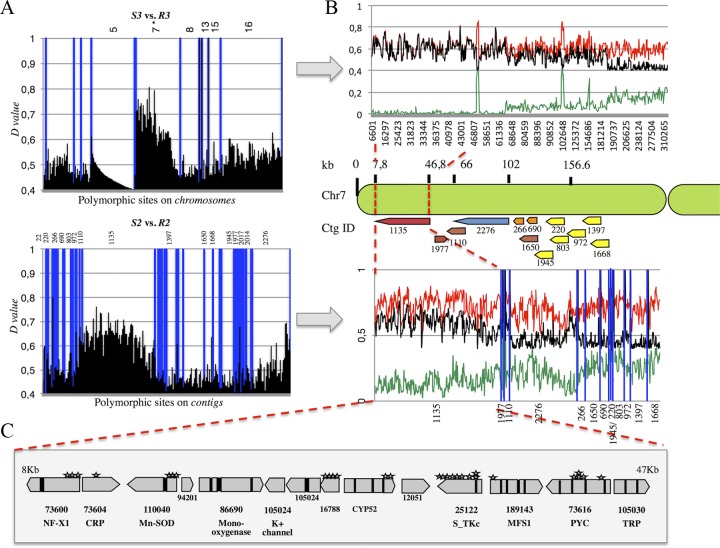
Polymorphism discovery in 09-CB1 × IPO323 versus 09-ASA-3apz × IPO94269 bulks.** (**A) *D* values (GQ_R_ − GQ_S_) plotted against their chromosomes (where IPO323 represents the sensitive parent) or contigs (where IPO94269 represents the sensitive parent) derived from 09-CB1 × IPO323 (upper panel) and 09-ASA-3apz × IPO94269 (lower panel). (B) Incrementing the bin size resolution of the regions concerned by the highest distortion, which shows a net increase of the *D* values (black line) between resistant bulk’s (red lines) and sensitive bulk’s GQ scores. Contigs showing the highest distortion (containing at least one polymorphic site with a *D* value of ≥0.5) from the 09-ASA-3apz × IPO94269 bulk sequencing were selected and aligned against the IPO323 genome. (C) Contig 1135 matching the region extending from 8 kb to 47 kb of chromosome 7 with the highest *D* values harbors 14 genes. SNPs and indels inducing nonsynonymous substitutions in the coding regions are indicated by stars.

Reads derived from each bulk and from the MDR parental strains were mapped on their respective reference sequences for IPO323 (R3 and S3 reads) and IPO94269 (09-ASA-3apz, R2 and S2 reads). The mapping procedure yielded comparable numbers of polymorphic sites (more than 180,000) and densities for both data sets (see Table S1 in the supplemental material). Single nucleotide polymorphism (SNP) and indel frequencies relative to each reference sequence were calculated and reported as genotype quality (GQ) values of between 0 and 1 (100%). We assumed that the GQ value of the region surrounding the *mdr* mutations would tend toward 1 in the resistant bulks, whereas the GQ values at the same sites would approach 0 in the sensitive bulks. By applying the thresholds GQ of ≥0.5 to the resistant bulks and GQ of ≤0.5 to the sensitive bulks and considering as relevant only sites with a maximum difference (*D* value) between both bulks (GQ_R_ − GQ_S_ = *D* value of ≥0.4), we were able to reduce the bin size around the distortion to several kilobases (Fig. 2B) located on the left arm of chromosome 7 of IPO323. In the 09-CB1 × IPO323 data set, this region showed the highest distortion, decreasing from the telomere to the centromere (Fig. 2B). The strategy was merely the same in the 09-ASA-3apz × IPO94269 data set, except for the use of unassembled contigs. Thirteen contigs out of 56 with *D* values of >0.4 colocalized on the left arm of chromosome 7 as well (Fig. 2B), out of which contig 1135 had the highest number of polymorphic sites with the highest *D* values. We therefore focused our subsequent analysis on the region of chromosome 7 covered by this contig (from positions 8 to 47 kb in Fig. 2C). Reporting all SNPs and indels of the resistant bulks R2 and R3 present in this bin on the local alignment between contig 1135 and the IPO323 very left arm of chromosome 7, we identified three kinds of polymorphic sites: those common to both MDR strains and those independent to each of them (see Table S3 in the supplemental material). The highest detected *D* values were, respectively, recorded in the *CYP52* gene (R2 and R3, *D* value = 0.72 [synonymous substitution]), in the pyruvate carboxylate gene *PYC* (R2; *D* value = 0.738 [intron]), and the manganese-iron superoxide gene *Mn-SOD* (R3; *D* value = 0.736 [5′ untranslated region; 5′ UTR]). Intriguingly, this region also covers the transporter gene *MFS1*, whose involvement in drug efflux and MDR was shown before (26, 40). This gene is located between the above-mentioned polymorphic sites. In particular, the genes immediately surrounding *MFS1*, *STK*, and *PYC* harbored many polymorphic sites that cosegregated with the MDR phenotype, while the 5′ UTR and 3′ UTR of the *MgMFS1* gene appeared structurally highly dissimilar in the 09-ASA-3apz and 09-CB1 backgrounds from both reference sequences, as stated by the dramatic decrease of mapped reads (data not shown). Indeed, we had previously found that both strains harbor a 519-bp insert in the *MFS1* promoter region (26).

10.1128/mSphere.00393-17.2 TABLE S1  Reads and mapping descriptive statistics of BSA. Download TABLE S1, XLSX file, 0.4 MB. Copyright © 2017 Omrane et al. 2017 Omrane et al. This content is distributed under the terms of the Creative Commons Attribution 4.0 International license.

Altogether, this BSA revealed for both MDR strains a region of 39 kb on chromosome 7 whose polymorphism strongly cosegregated with the MDR phenotypes. Since the cross between both MDR strains indicated that the responsible mutations are allelic or closely linked, the identification of this common region from both independent BSA experiments is in favor of its involvement in the MDR phenotype.

In order to precisely map the *mdr* mutation on the 39-kb fragment of 09-CB1, we analyzed the alleles of the marker genes at each extremity of the region by high-resolution melt (HRM) analysis, as well as the *MFS1* promoter genotype by PCR in all progeny strains derived from the cross between 09-CB1 and IPO323. Table 2 shows that only the *MFS1* promoter genotype of the MDR parental strain strictly cosegregated with the MDR phenotype. A 100% cosegregation between the *MFS1* promoter allele and the MDR phenotype was also observed for all 297 progeny strains derived from cross 2 between 09-ASA-3apz and IPO94269.

**TABLE 2:** Genotyping of progeny strains for *MFS1* alleles and linked marker genes

Cross and marker gene	No. of strains^a^			
	MDR progeny		Sensitive progeny	
09-CB1 × IPO323	09-CB1	IPO323	09-CB1	IPO323
* NFX1*	96	1	1	110
* PYC*	95	2	1	110
* MFS1*	97	0	0	111
09-ASA-3apz × IPO94269	09-ASA-3apz	IPO94269	09-ASA-3apz	IPO94269
* NFX1*	NA	NA	NA	NA
* PYC*	NA	NA	NA	NA
* MFS1*	148	0	0	149

a For cross 09-CB1 × IPO323, *n* = 97 MDR progeny and *n* = 111 sensitive progeny strains. For cross 09-ASA-3apz × IPO94269, *n* = 148 MDR progeny and *n* = 149 sensitive progeny strains. NA, not analyzed.

**FIG 3:**
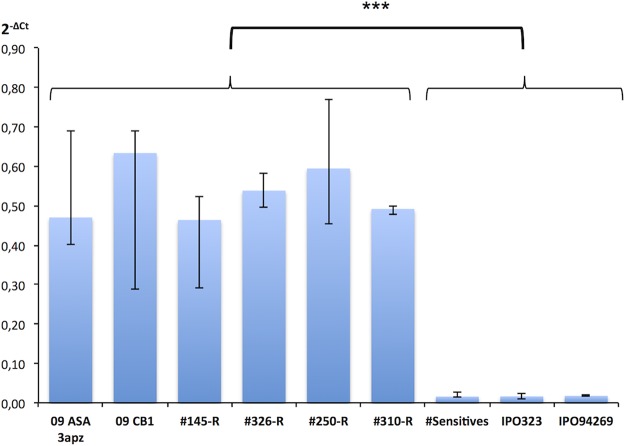
*MFS1* expression in parental and progeny strains. *MFS1* expression was measured by qRT-PCR relative to three reference genes (β*-tubulin*, *eF1a*, and *actin*). Median values (*n* = 4 to 12) and interquartile range are indicated in the plot. Progeny strains with an “R” suffix are of the MDR phenotype. The value plotted as “#Sensitives” corresponds to expression data from six independent sensitive strains (*n* = 12). ***, significant difference of the *MFS1* expression levels between MDR and sensitive strains according to Kruskall and Wallis’ nonparametric statistical test with a risk threshold at 0.1%.

Expression analysis of *MFS1* in selected offspring showed *MFS1* overexpression in resistant progeny strains (145-R, 326-R, 250-R, and 310-R) comparable to that in the MDR parental strains, while the “sensitive” strains shown in Fig. 3 displayed the basal expression level characteristic of sensitive strains.

### Functional validation of the identified *mdr* mutation (*MFS1* insert).

**FIG 4:**
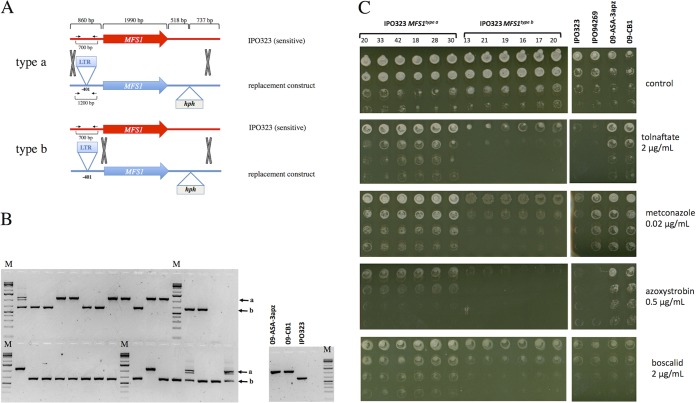
Functional analysis of the *MFS1*^*MDR*^ allele in the sensitive IPO323 strain. (A) Principle of possible homologous recombination events at the *MFS1* locus with the replacement construct. The gray X’s indicate homologous recombination events leading to the integration of the hygromycin resistance marker gene *hph*. The black arrows indicate the positions of the primers 2F and 4R used to distinguish between type a and type b recombination events. (B) PCR analysis of isolated transformants with primers 2F and 4R. The black arrows indicate the bands produced by type a or type b recombination. M, molecular size markers. (C) Growth tests of selected transformants on fungicides with different modes of action. Serial dilutions (from top to bottom rows) of calibrated precultures of the indicated strains were inoculated on growth test plates (YPD with the indicated fungicides) and incubated at 17°C for 5 days.

In order to validate the involvement of the 519-bp promoter insert in the MDR phenotype, we proceeded through the replacement of the wild-type MFS1 allele (*MFS1*^*WT*^) by the *MFS1*^*MDR*^ allele in the sensitive reference strain IPO323 (Fig. 4A and B). After selection and isolation on hygromycin, transformants were screened by PCR analysis with primers Z4_110044_FW and Z4_110044_RV (see Table S2 in the supplemental material) flanking the promoter insert (Fig. 4A and B), to discriminate those obtained by integration of the replacement construct at the *MFS1* locus from those with ectopic integration, as the latter showed more than one amplicon. Moreover, this PCR distinguished between integration events of type a and type b: i.e., type a integration led to the promoter insert, while type b integration did not (Fig. 4A).

10.1128/mSphere.00393-17.3 TABLE S2  Primers used in this study. Download TABLE S2, DOCX file, 0.1 MB. Copyright © 2017 Omrane et al. 2017 Omrane et al. This content is distributed under the terms of the Creative Commons Attribution 4.0 International license.

10.1128/mSphere.00393-17.4 TABLE S3  SNP data of BSA. Download TABLE S3, XLS file, 2.6 MB. Copyright © 2017 Omrane et al. 2017 Omrane et al. This content is distributed under the terms of the Creative Commons Attribution 4.0 International license.

**FIG 5:**
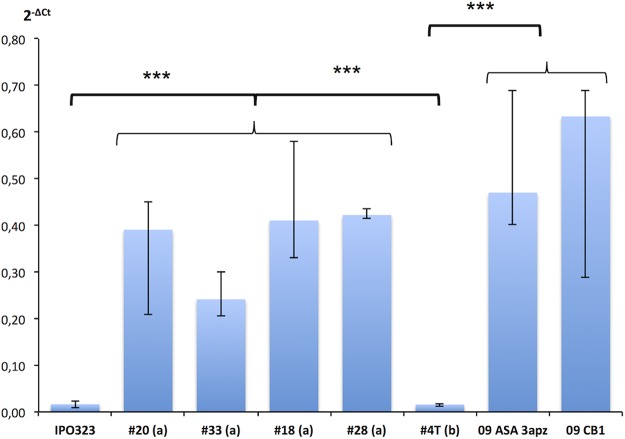
*MFS1* expression in IPO323 *MFS1* replacement mutants. *MFS1* expression was measured by qRT-PCR relative to three reference genes (β*-tubulin*, *eF1a*, and *actin*). Median values (*n* = 4 to 8) and interquartile range are indicated in the plot. Transformants are indicated by the symbol #. The letters in parentheses refer to the recombination event in Fig. 4. A type a recombination leads to the promoter insert of 519 bp and the MDR phenotype, while a type b recombination event does not. #4T corresponds to expression data from four independent transformants with a type b recombination event (*n* = 8). ***, significant difference of the *MFS1* expression levels between strains or groups of strains according to Kruskall and Wallis’ nonparametric statistical test with a risk threshold at 0.1%.

Six transformants of each category were tested for growth on different fungicides. As seen from Fig. 4C, only the integration of the 519-bp insert in the *MFS1* promoter (type a integration) conferred increased tolerance to the squalene epoxidase inhibitor tolnaftate, the DMI metconazole, the quinone outside inhibitor (QoI) of cytochrome bc1, azoxystrobin, and the SDHI boscalid. Similar results were observed with terbinafine, prochloraz, and bixafen (data not shown). Type b integrants displayed the same sensitivity to the compounds as the parental IPO323 strain. Four selected transformants with the promoter insert (transformants 20, 33, 18, and 28) overexpressed *MFS1* compared to IPO323, while type b transformants (without the insert) did not (Fig. 5).

We may thus conclude that the 519-bp LTR insert in the *MFS1* promoter leads to its overexpression and consequently to an MDR phenotype.

### *MFS1* promoter and expression analysis in *Z. tritici* field isolates.

In a population survey, we tested if the *MFS1* promoter insert was present in all field isolates with the phenotype MDR, applying PCR with the primer pair MFS1_2F/MFS1_4R (Table S2). No insert was detected in non-MDR strains. In 35 genotyped MDR strains, however, we obtained three different amplicons of 1,000, 850, and 650 bp, respectively, corresponding to three different inserts, designated type I, II, and III inserts, while no insert was detected in over 100 non-MDR field strains (M. Garnault and A.-S. Walker, unpublished data). The 519-bp LTR insert (type I) was the most frequent insert detected since 2009, the type II insert was detected in 25% of the strains and had been present since 2012, and the type III insert was detected in only two strains from 2015 (Garnault et al., unpublished).

We determined the 50% effective concentration (EC_50_) values on the selected DMIs terbinafine and tolnaftate for field strains representative of each type of insert (*n* = 7 for type I, *n* = 5 for type II, and *n* = 2 for type III). Resistance factors of the three genotypes to tolnaftate and terbinafine (unlinked to any specific resistance and not affected by the *CYP51* alleles), listed in Table 3, reveal similar phenotypes for strains with type I and II inserts, while the resistance factor conferred by the type III insert seems weaker.

**TABLE 3:** Resistance factors of *Z. tritici* field isolates with different *CYP51* and *MFS1* alleles relative to the *CYP51*^*WT*^* MFS1*^*WT*^ genotype^b^

Antifungal	EC_50_ (ng ml^−1^) for *CYP51*^*WT*^* MFS1*^*WT*^	RF for^a^:			
		*CYP51^TriR^ MFS1^WT^*	*CYP51^TriR^ MFS1^MDR type I^*	*CYP51^TriR^ MFS1^MDR type II^*	*CYP51^TriR^ MFS1^MDR type III^*
**Tolnaftate**	**15 ± 3.5**	**ND**	**11.9**	**11.5**	**3.8**
**Terbinafine**	**0.1 ± 0.02**	**ND**	**84.3**	**87.6**	**37.5**
Epoxiconazole	0.35 ± 0.17	258	959	1,138	927
Prothiocon-azole- desthio	0.32 ± 0.18	51.9	73.6	107	144
Tebuconazole	3.4 ± 0.9	609	546	381	269
Metconazole	0.21 ± 0.04	360	655	572	1,116
Pyrifenox	0.38 ± 0.14	559	610	1,054	709

a The *CYP51*^*TriR*^ genotype groups together different *CYP51* genotypes of field strains with cross-resistance to DMIs. The resistance factors (RFs) are expressed as the EC_50_ of the studied genotype/EC_50_ of the *CYP51*^*WT*^* MFS1*^*WT*^ genotype. ND, not determined.

b RFs relative to tolnaftate and terbinafine are affected only by the different MFS1 alleles (no target site mutations). Therefore, these values are in boldface.

**FIG 6:**
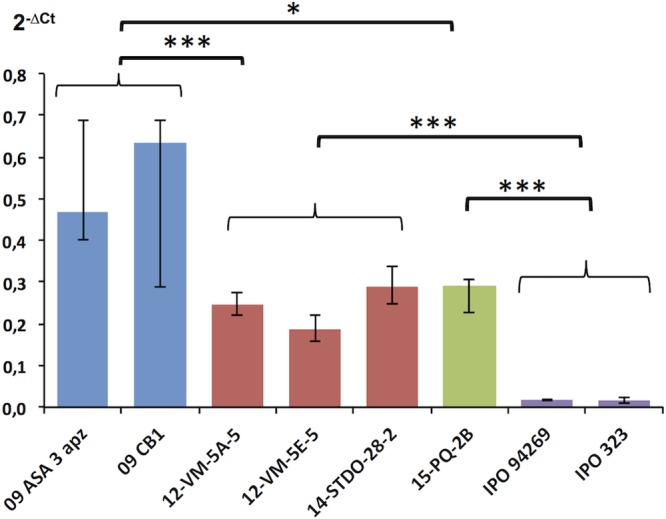
*MFS1* expression in *Z. tritici* field isolates with different promoter genotypes. *MFS1* expression in selected field strains harboring type 1 to 3 inserts (blue, type I; red, type II; green, type III; violet, no insert). *MFS1* expression was measured by qRT-PCR relative to three reference genes (β*-tubulin*, *eF1a*, and *actin*). Median values (*n* = 4 to 10) and interquartile range are indicated in the plot. ***, significant difference of the *MFS1* expression levels between strains or groups of strains according to Kruskall and Wallis’ nonparametric statistical test with a risk threshold at 0.1%; *, significant differences at a risk threshold of 5%.

The *MFS1* transporter gene was found to be overexpressed as well in the field strains with type II and type III inserts (Fig. 6), although at significantly lower levels than in strains with the type I insert.

### Sequence of the *MFS1* promoter in *Z. tritici* MDR field isolates.

**FIG 7:**
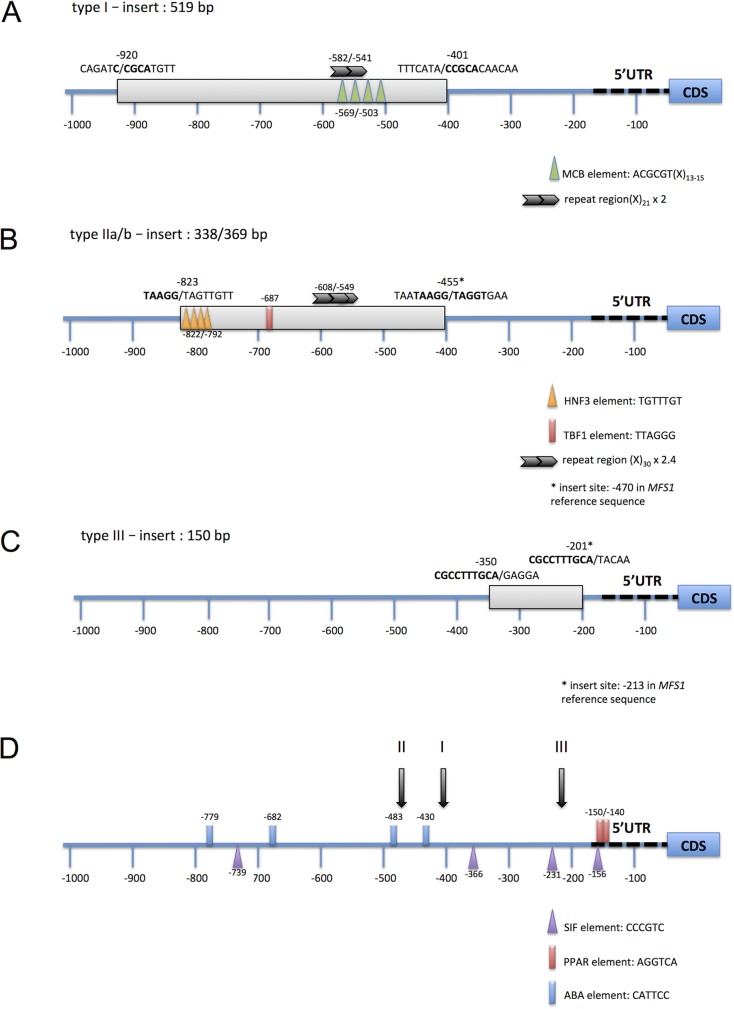
Molecular structure of *MFS1* promoter genotypes. The figures indicate the location, length, and insert sites of *MFS1* promoter inserts. Repeats between both insertion sites are indicated by boldface letters. Consensus sequences of potential regulatory elements identified by TRANSFAC searches (44) are indicated by the colored triangles and boxes. Repeats of longer sequences, found using mreps (45), are indicated by the black arrows. The MCB element (MluI cell cycle box) regulates the expression of genes involved in G_1_-phase transition (46). HNF3 is a *Rattus norvegicus* motif (87, 88). The hexamer TTAGGG corresponds to the *S. cerevisiae* TBF1 element (89).

We have sequenced the region 500 bp upstream of the *MFS1* start codon in 26 MDR field isolates (GenBank accession no. MF623010 to MF623033) (see Fig. S1 in the supplemental material). This region covers the three types of inserts that localize in a region between 200 and 500 bp upstream of the start codon (Fig. 7D). The integration sites all displayed short repeated sequences (5 to 10 bp). The type II insert was found in two lengths: 369 bp (type IIa) and in a 30-bp shorter version (type IIb) that was otherwise 87% identical. Type III inserts were identical between both strains: 149 bp in length.

10.1128/mSphere.00393-17.1 FIG S1  Sequence alignment of the *MFS1* promoter of *Z. tritici* field strains. Download FIG S1, PDF file, 0.2 MB. Copyright © 2017 Omrane et al. 2017 Omrane et al. This content is distributed under the terms of the Creative Commons Attribution 4.0 International license.

As mentioned earlier, the type I insert corresponds to the 519-bp LTR sequence of a Ty1/Copia retrotransposon localized on chromosome 18 (26) in the sequenced strain IPO323 and annotated as still active (36, 41).

We also screened the sequence of the type II insert against available *Z. tritici* sequences by BLASTn searches (42). The query sequence got multiple hits against the IPO323 sequence (51 hits with >95% identity over >200 bp), had three matches on the genome sequence of JGIBBWB-9N22, and was found 99% identical to the insert found upstream of *CYP51* in some *Z. tritici* DMI-resistant isolates, in particular in strain 9-ASA-3apz (26). These results indicate that the type II insert may be a repeated element of the *Z. tritici* genome or part of it.

Searching the sequence of the type III insert against the IPO323 genome sequence also gave five hits with >86% identity over more than 110 bp with unannotated nucleotide sequences.

### Identification of putative regulatory elements in the *MFS1* promoter.

Since the type I insert drives constitutive overexpression of the *MFS1* gene, we suspected this element to contain upstream activation sequences (UASs). UASs may act to recruit transcription activators or coactivators, increasing the affinity of the general transcription machinery or through opening the chromatin structure (reviewed in reference 43), leading to higher transcription levels. We searched for known transcription factor binding sites of the TRANSFAC database (44) as well as for larger tandem repeats or palindromic repeats using mreps (45). As highlighted in Fig. 7A, we identified four successive consensus sequences of the MCB element (MluI cell cycle box, also termed an MBP element [ACGCGT]), separated by 13 to 15 bp, respectively, in a region 569 to 503 bp upstream of the start codon. This region also overlaps a tandem repeat of 21 bp (positions −582 to −541). The MCB element is a hexamer sequence regulating the expression of genes involved in G_1_-phase transition, first identified in *S. cerevisiae* (46). The number of MCB repeats correlates with the expression level (47). As MCB elements are well conserved in the promoters of G_1_-phase genes among fungal species (48), one may suspect that the four elements present in the type I insert drive *MFS1* expression in strains harboring this insert.

The same strategy was applied to identify putative UASs in type II and type III inserts. While in the shortest insert (type III) no repeated sequence was identified, the type II insert harbors different potential regulatory elements, as highlighted in Fig. 7B.

The original *MFS1* promoter of the sensitive IPO323 strain contains four repeats of the *Aspergillus nidulans* AbaA binding site CATTCC (49), 779 to 430 bp upstream of the start codon, four repeated SIF elements (50, 51), and two adjacent peroxisome proliferator-activated receptor (PPAR [or PPRE]) elements (52) in the 5′ UTR. Some of these elements may be involved in the regulation of *MFS1* transcription under fungicide treatment (26) or under yet unknown conditions.

## DISCUSSION

Increased drug tolerance through increased efflux is a general phenomenon threatening major clinical treatments, including anticancer, antibacterial, and antifungal drugs, respectively (9, 13, 53, 54). In agriculture, the same phenomenon resulting in MDR has been documented in the last decades with the identification of the membrane transporters involved (17, 20–22, 26, 55). While drug efflux *per se* does not confer high resistance levels, the combination with specific resistance mutations can strongly impair the efficacy of agrochemical fungicides. This phenomenon is already being observed in *B. cinerea* (22, 23, 55), *S. homeocarpa* (19, 20), with several modes of action, and, in the case of *Z. tritici*, with DMIs (8). The introduction of new modes of action will help dealing with the diseases despite complicated resistance situations, but intelligent treatment strategies must be applied to delay and limit the risk of development and recombination with already fixed resistances.

The mutations responsible for clinical antifungal MDR identified so far are principally gain-of-function mutations in the *C. albicans* transcription factors CaTac1 and CaMrr1 and the *C. glabrata* factor CgPdr1 regulating the expression of ABC or MFS transporter genes (reviewed in references 13 and 15). In the plant-pathogenic fungus *Botrytis cinerea*, similar gain-of-function mutations in the transcription factor BcMrr1 have been identified in MDR field strains, responsible for the overexpression of the ABC transporter-encoding gene *BcatrB* (22, 23). In addition, the mutation leading to the overexpression of the MFS transporter-encoding gene *BcmfsM2* was identified as a promoter deletion-insertion event, the insert being a retroelement-like gene fragment (22).

In our previous study, we identified MFS1 as a major player in *Z. tritici* multidrug resistance (26) and had found a retrotransposon relic as an insert in the *MFS1* promoter. In this study, we used an unbiased approach by classical and high-throughput genetics to identify the MDR-responsible mutations.

We discovered that the *mdr* mutations of both analyzed strains are identical, although their MDR phenotypes differ slightly (8) and their drug efflux is affected differently by chemical modulators (26). Therefore, one may suspect additional mutations explaining such differences. The observed segregation between sensitive versus MDR strains among the progeny was close to the ratio of 1:1, but the selection of offspring was not completely random. A potential bias can have its origin in the analyzed progeny, as we eliminated 25% of the offspring as mixtures or impure strains. In addition, the phenotypic screen for the MDR phenotype may have been too stringent to identify slight quantitative differences in resistance to tolnaftate that could have originated from multiple quantitative mutations.

The present study identified three different types of inserts in the *MFS1* promoter region potentially involved in *MFS1* overexpression and, consequently, in MDR. The most frequent insert, type I, is the previously identified relic of a still active Ty1/Copia retrotransposon (41); the type II insert also resembles a repetitive element, as it was detected at >100 instances in the *Z. tritici* genome sequence. Also the type III insert was found repeated, but less frequently. We have shown that the LTR insert (type I) is responsible for *MFS1* overexpression through classical and reverse genetics. Using a similar gene replacement strategy, we could also confirm the role of inserts II and III in resistance to tolnaftate and presumably in MDR (data not shown).

We addressed the question of whether the inserts drive *MFS1* expression on their own or if they disrupt transcription repression, through *in silico* analysis of the generated promoter sequences. In the case of the LTR insert, it is highly probable that the insert drives *MFS1* expression on its own, as LTR elements harbor *cis-*regulatory sequences (reviewed in references 56 and 57). The *MFS1* type I LTR element harbors four potential MCB elements known as sequence regulating expression of G_1_-phase genes in *S. cerevisiae* (46, 58). As the sequence of this element was found highly conserved upstream of G_1_-phase genes in fungi (48), these elements probably drive the strong constitutive expression of *MFS1* in type I MDR strains. Moreover, the regulation of transcription according to cell cycle progression by MCB elements may explain the strong variation observed in the *MFS1* expression levels in type I MDR strains. Potential UASs were also detected in the type II insert, eventually driving *MFS1* constitutive overexpression, while the type III insert seems devoid of novel regulatory elements. In sensitive *Z. tritici* strains, *MFS1* expression is induced after fungicide challenge (26), while all inserts lead to high basal expression. These results suggest that the LTR insert drives expression on its own while abolishing the induction under fungicide challenge (26). The function of type II insert might be similar (induction instead of release of repression), while in the case of the type III insert, because of its position close to the 5′ UTR and the absence of known UASs, we may suspect that this element rather releases the inhibition of *MFS1* transcription. To fully understand the regulation of MFS1 expression and discriminate between both hypotheses, the role of the potential regulatory elements in *MFS1* transcription regulation remains to be established through promoter fusion experiments. In addition the transcription regulators involved in *MFS1* regulation in response to drug challenge remain to be identified in *Z. tritici*.

Finally, our analysis of *Z. tritici* field strains revealed three to four different insertion events into the *MFS1* promoter with strong impact on the fungicide sensitivity phenotype. The use of mobile elements mediating fungicide resistance through target gene overexpression is a common mechanism among phytopathogenic fungi. Especially in the *CYP51* promoters of various fungal species, repeated elements or relics of them were found: e.g., *Penicillium digitatum* (59, 60), *Blumeria jaapii* (61), *Monilinia fructicola* (62), *Venturia inequalis* (63, 64), and *Z. tritici* (65). Transposable elements (TEs) are known to modify genome structure, gene functions, and phenotypes (56, 66–68). In filamentous fungi, the genome content in TEs can be highly variable even between closely related species (57, 69). TEs are suspected to contribute to host adaptation (e.g., in *Magnaporthe oryzae* [70]), speciation, and higher adaptive capacity to selective pressure (including pathogenicity evolution) by generating genomic rearrangements (69, 71). It was recently established that >17% of the *Z. tritici* genome sequence was repetitive, out of which 70% corresponds to retrotransposable elements (41, 72).

Among the LTR transposons identified by Dhillon et al. (41), one (family 18) showed minimal evidence of repeat induced point mutations (RIP) and was therefore supposed to be still active. Indeed, the LTR sequence corresponding to the MFS1 type I insert is 100% identical to this family 18 LTR. We may therefore suspect that this LTR insertion is due to a recent retrotransposition event. According to their data, Grandaubert and coworkers supposed that class II DNA transposons also are still active in *Z. tritici* (72). They might be at the origin of the other two insertion events.

Little is known about the factors inducing (retro)transposon mobilization in current populations, nor about the impact of fungicide exposure on this phenomenon. Chen and colleagues observed increased mobilization of the transposable element *Mftc1* in *M. fructicola* after *in vitro* exposure to sublethal fungicide concentrations, although not affecting fungicide sensitivity (73).

The question remains of whether the *MFS1* promoter is prone to insertion events relatively more frequently than other genomic loci or if this is only due to fungicide selection pressure. The available and forthcoming *Z. tritici* whole-genome sequences and associated transcriptomic data will shed light on not only the influence of transposable elements on genome structure and global gene expression (72, 74, 75) but also their evolution. Population genomics and experimental evolution may also help in evaluating the impact of fungicide pressure on transposon mobilization and subsequent selection of insertion events.

## MATERIALS AND METHODS

### *Z. tritici* strains and growth conditions.

All *Z. tritici* field strains used in this study are listed in Table 4. As sensitive reference strains, we used IPO323 and IPO94269, displaying *MAT1-1* and *MAT1-2* genotypes, respectively (76), and sensitive to all tested fungicides (40) (our unpublished results). As principal MDR strains, we used 09-ASA-3apz and 09-CB01 (8, 26). Strains produced in this study are described below. All strains (if not otherwise indicated) were cultivated on solid YPD medium (20 g liter^−1^ bacteriological peptone, 10 g liter^−1^ yeast extract, 20 g liter^−1^ glucose, 15 g liter^−1^ agar) for 7 days at 17°C in the dark. The sequence of IPO323 is publically available (36), and that of IPO94269 was kindly provided by Syngenta AG for mapping analysis.

**TABLE 4:** *Zymoseptoria tritici* field strains used in this study

Strain	Fungicide sensitivity profile^a^	*MFS1* insert
IPO323	Sensitive	None
IPO94269	Ben^r^	None
09-ASA-3apz	Tri^r^ Str^r^ Ben^r^ MDR	Type I
09-CB1	Tri^r^ Str^r^ Ben^r^ MDR	Type I
SYN20	Sensitive	None
07-S6	Sensitive	None
07-S46	Sensitive	None
SYN33	Sensitive	None
14-AK-1a	Tri^r^ Str^r^ Ben^r^	None
15-OM-4A	Tri^r^ Str^r^ Ben^r^	None
12-AGC-13C	Tri^r^ Str^r^ Ben^r^	None
12-VM-4F	Tri^r^ Str^r^ Ben^r^	None
ST-5548	ND	None
14-AQ-1	Tri^r^ Str^r^ Ben^r^	None
14-STIRLO-13	Tri^r^ Str^r^ Ben^r^ Car^r^	None
15-PN-3	Tri^r^ Str^r^ Ben^r^ Car^r^	None
STDP-04915	Tri^r^ Str^r^ Ben^r^ Car^r^	None
15-OU-1D	Tri^r^ Str^r^ Ben^r^	None
15-PQ-8B	Tri^r^ Str^r^ Ben^r^ MDR	Type III
15-PQ-2B	Tri^r^ Str^r^ Ben^r^ MDR	Type III
14-MAFL-08	Tri^r^ Str^r^ Ben^r^ MDR	Type II
14-AK-1b	Tri^r^ Str^r^ Ben^r^ MDR	Type II
12-VM-4J	Tri^r^ Str^r^ Ben^r^ MDR	Type II
14-STDO.28.2	Tri^r^ Str^r^ Ben^r^ Car^r^ MDR	Type II
12-VM-5A	Tri^r^ Str^r^ Ben^r^ MDR	Type II
12-VM-5C	Tri^r^ Str^r^ Ben^r^ MDR	Type II
12-VM-5F	Tri^r^ Str^r^ Ben^r^ MDR	Type II
12-VM-5E	Tri^r^ Str^r^ Ben^r^ MDR	Type I
13-AHJ-8C	Tri^r^ Str^r^ Ben^r^ MDR	Type I
14-EG-A1	Tri^r^ Str^r^ Ben^r^ MDR	Type I
15-OM-5	Tri^r^ Str^r^ Ben^r^ MDR	Type I
12-VM-7A	Tri^r^ Str^r^ Ben^r^ MDR	Type I
10-BNE35	Tri^r^ Str^r^ Ben^r^ MDR	Type I
14-STDK	Tri^r^ Str^r^ Ben^r^ MDR	Type I
15-PQ-6A	Tri^r^ Str^r^ Ben^r^ MDR	Type I
09-ASA-10bpz	MDR^b^	Type I
12-VM7A	Tri^r^ Str^r^ Ben^r^ MDR	Type I

a The resistance phenotypes are indicated as follows: Tri^r^, triazole resistance (any phenotype specifically resistant to DMIs); Str^r^, strobilurin resistance; Ben^r^, benzimidazole resistance; Car^r^, carboxamide resistance; MDR, multidrug resistance. Establishment of phenotypes was performed as published in reference 8. ND, not determined.

b Specific resistance phenotypes not determined.

To determine EC_50_s to prothioconazole-desthio, tebuconazole, metconazole, tolnaftate, terbinafine, and pyrifenox, conidia were collected from 3-day-old cultures on NY medium (2 g/liter malt, 2 g/liter yeast extract, 15 g/liter agar) in sterile water and adjusted to a final concentration of approximately 2 × 10^5^ conidia ml^−1^. Three hundred microliters of each solution was spread on test plates containing solid phosphate-glucose medium (2 g liter^−1^ K_2_HPO_4_, 2 g liter^−1^ KH_2_PO_4_, 10 g liter^−1^ glucose, 12.5 g liter^−1^) with 5-fold serial dilutions (2.5-fold) of prothioconazole-desthio (Sigma-Aldrich, Saint Quentin Fallavier, France) tebuconazole (technical grade; Bayer, CropScience, Germany), metconazole (technical grade; BASF Agro, Germany), tolnaftate (Sigma-Aldrich), terbinafine (technical grade; Sandoz, Switzerland) and pyrifenox (technical grade; Syngenta Agro, Switzerland) as described in reference 8. All fungicides were supplied as 250× concentrated ethanol solutions. Test and control plates were incubated at 17°C in the dark for 48 h. The length of the germ tube was estimated microscopically on 10 to 30 spores per plate. The EC_50_ of each tested fungicide corresponding to the concentration inhibiting spore germination by 50% was determined by nonlinear regression (least-square curve fitting) using the GraphPad PRISM program (GraphPad Software, Inc., La Jolla, CA).

### Crosses and progeny phenotyping.

Crosses 1 (09-ASA-3apz × 09-CB1), 2 (09-ASA-3apz × Ipo94269), and 3 (09-CB1 × Ipo323) were performed as described previously (37). Single spore progenies were isolated from asci. A set of 20 progeny strains from each cross were checked by genotyping of 11 simple sequence repeats (SSRs) on the core chromosomes (77) in comparison to their parents to validate the absence of external contaminants prior to all subsequent analyses. The whole offspring was genotyped using a set of eight SSRs. Only strains presenting single bands were conserved.

Sensitivity tests to distinguish MDR from sensitive offspring were performed in solid as well as in liquid YPD medium with the addition of tolnaftate (2, 5, or 10 µg ml^−1^). All strains were grown on solid YPD for 1 week (18°C in continuous light) and transferred by toothpicks to a 96-well microtiter plate in 200 µl sterile water. Ten microliters of 1/100 dilutions was spotted onto 96-well microtiter plates filled with YPD (liquid) with and without tolnaftate. The plates were incubated on a rotary shaker at 18°C during 11 days. Scoring of growth was made by OD measurement (λ = 590 and 620 nm) at 3, 6, and 11 days. Growth rates at days 6 and 11 were calculated relative to day 3 and as a ratio of treated versus untreated conditions. The whole assessment procedure was repeated three times.

### Progeny bulk preparation.

Resistant (MDR phenotypes) and sensitive sets (sensitive phenotypes) of strains were selected on the basis of the phenotyping described above among those that grew or not on 5 µg ml^−1^ of tolnaftate to build bulks for each of the progenies (crosses 2 and 3) before nucleic acid extraction. The numbers of strains for the DNA bulks were, respectively, *n* = 60 for R2 and S2 (resistant and sensitive bulks, respectively, from cross 2) and *n* = 50 for R3 and S3 (resistant and sensitive bulks, respectively, from cross 3). The phenotype of all selected strains was verified on tolnaftate (5 µg ml^−1^) by growth tests on solid medium and liquid medium. Additionally, liquid growth tests were performed with or without the addition of reversal agents, namely, amitriptyline, chlorpromazine, and verapamil (Sigma-Aldrich) at a 1:3 ratio (5 µg ml^−1^tolnaftate to 15 µg ml^−1^reversal agents).

For DNA extraction, each strain was grown individually in liquid YPD for 1 week on a rotary shaker (140 rpm at 18°C). Cultures were harvested by centrifugation (4,000 rpm for 10 min), rinsed twice with cold 1× phosphate-buffered saline system (PBS [Sigma-Aldrich]), and immediately frozen in liquid nitrogen prior to vacuum freeze-drying. DNA samples were extracted from freeze-dried cells using the DNeasy Plant maxi kit (Qiagen, Courtaboeuf, France). The concentrations were determined and the quality verified on a 2100 Bioanalyzer (Agilent). Identical amounts (200 ng) of genomic DNA of each strain were mixed to constitute the R2, S2, R3, and S3 bulks.

### Sequencing and mapping to reference genome.

100 base paired-end Illumina sequencing was performed on 2 µg of DNA of each bulk as well as for the MDR progenitors by the sequencing provider (Beckman-Coulter, Takeley, United Kingdom). The total number of reads used for genome mapping was 32,269.4 Mb, distributed equally among the DNA samples (Table S1). These were mapped, respectively, on reference genome sequences of the sensitive parental strains IPO94269 (reads from 09-ASA-3apz and R2 and S2 bulks) or IPO323 (09-CB1 and R3 and S3 bulks) by the sequencing provider, using Bwa 0.6.1 (78) for alignment and the GATK GenomeAnalysis module (79) for local realignment. Variants were called with Samtools 1.18 (80) using default parameters. The GATK Unified Genotyper (79) was used to call all locations of allele frequencies. For 09-CB1 and R3 and S3 bulks, snpEff 3.0 was used to call effects for filtered variants. Finally, VarSifter 1.5 (81) was used to inspect final genotype calls for coherence. IGV 2.3 (82) was used to visualize the polymorphisms (SNPs and indels) per cross project, respectively. For cross 2, only contigs of IPO94269 over 200 bp were used as reference sequences. For cross 3, the JGI Mycgr3 genome (http://genome.jgi.doe.gov/Mycgr3/Mycgr3.home.html) was used together with the “FrozenGeneCatalog20080910” gene predictions.

Alignments of both reference sequences were performed by MAUVE (83). The reads’ quality cutoff and read depth were set to ≥20 and ≥5, respectively. The final filtering parameters to detect the genomic distortions were set as GQ scores (encoded as a phred quality) for each genotype as ≥0.5 for resistant bulks and <0.5 for sensitive bulks, while the GQ scores for the parental genotypes were set as 1 for MDR and 0 for the sensitive reference sequence. The difference between R and S bulk genotypes (*D* value) was filtered as ≥0.4. BLASTn analyses of contig sequences were made at the Joint Genome Institute (JGI; https://genome.jgi.doe.gov/Mycgr3/Mycgr3.home.html) using the *M. graminicola* v2.0 unmasked nuclear assembly as the search criterion.

### Genotyping of progeny strains. (i) *MFS1* promoter.

Screening for the 519-bp insert in the *MgMFS1* promoter (26) among all progeny strains was performed by PCR using the primer pair MFS1_2F and MFS1_4R (Table S2). No promoter insert led to a 486-bp amplicon, while the insert increased the amplicon size to 1,005 bp.

### (ii) *NFX1* and *PYC* polymorphism.

The genotypes of the *NFX1* and *PYC* genes located at both ends of contig 1135 of the IPO94269 genome sequence were determined on the progeny strains by HRM analysis in comparison to the parental strains. The primer pairs NFX1_11422FW/NFX1_11422RV and PYC_5UTR_FW/PYC_5UTR_RV (Table S2), showing close to 100% amplification efficiency on serial DNA dilutions of the parental strains, were used for HRM comparisons under the following conditions. In a total volume of 25 µl, 5 ng of genomic DNA was analyzed with 300 nM both primers using 1× SsoFast Evagreen Supermix (Bio-Rad, Marnes-la-Coquette, France). The cycling parameters were 98°C for 2 min followed by 40 cycles of 98°C for 2 s and 60°C for 5 s. The high-resolution melt curve was established between 70 and 95°C with 0.2°C increments every 10 s in a CFX-96 real-time PCR system (Bio-Rad). Melting curve normalization and differentiation were performed using Precision Melt Analysis software (Bio-Rad).

### *MFS1* expression analysis.

For analysis of *MFS1* expression during exponential growth, the tested strains (field strains, offspring, and transformants) were grown in liquid YPD (5 ml) at 18°C and 140 rpm for 48 to 72 h. The cell concentration was determined microscopically with a hemacytometer, diluted in 100 ml of YPD to a final concentration of 5 × 10^4^ cells/ml, and incubated for 48 h at 18°C at 140 rpm to a final cell concentration of 5 × 10^6^ cells/ml. The cultures were harvested by centrifugation (6,500 rpm at 4°C for 20 min), and the pellet was immediately frozen in liquid nitrogen and freeze-dried.

Total RNA was extracted from the freeze-dried mycelium using the RNeasy plant minikit (Qiagen) according to the supplied instructions. The quality of the RNA was checked by electrophoresis; the concentration was determined spectrophotometrically (NanoDrop; Thermo Scientific). One microgram of total RNA was used for cDNA synthesis using the PrimeScript RT reagent kit with gDNA Eraser (TaKaRa Bio, Inc., Saint-Germain-en-Laye, France). cDNAs were diluted 5 times before quantitative PCR (qPCR) analysis with the MESA green qPCR MasterMix Plus for SYBR assay (Eurogentec, Angers, France). The MFS1 amplicon was obtained with primers 110044_Fw and 110044_RV, which had been verified by standard curves for amplification efficiencies ranging from 95% to 105% and for the absence of nonspecific amplicons. Relative expression levels were determined according to the 2^−Δ*CT*^ threshold cycle method and the BestKeeper method (84) with *EF1α*, β*-tubulin*, and *UBC1* as the control genes to establish the most stable value of housekeeping gene expression among all experimental conditions. Medians and interquartile ranges were calculated from two technical replicates of at least three biological replicates. In the case of sensitive strains or transformants without a promoter insert, data obtained from four to six independent strains were grouped prior to statistics. All primer sequences are listed in Table S2.

Expression levels were statistically analyzed between strains or group of strains sharing common phenotypes (sensitive versus MDR) or a common *MFS1* genotype by nonparametric Kruskal-Wallis tests in multiple or pairwise comparisons. Significance thresholds were set at a risk α of 5% (*), 1% (**), or 0.1% (***) as indicated in Fig. 3, 5, and 6.

### *MFS1* gene replacement constructs.

To introduce the various *MFS1*^*MDR*^ alleles into the sensitive IPO323 strain, the following replacement cassettes were constructed. The respective *MFS1* allele, 1,380 bp upstream until 518 bp downstream of the open reading frame (ORF), was amplified from the corresponding DNA (09-ASA-3apz, 09-CB01, or other MDR strains) with the primer MDR-pKr_F at the 5′ end and the strain-specific primer MDR6_hygR (09-ASA-3apz) or MDR7_hyg R (09-CB01) at the 3′ end using Q5 high-fidelity DNA polymerase (New England Biolabs, Evry, France). A 737-bp 3′ flank of the *MFS1* gene to facilitate homologous recombination was amplified from IPO323 genomic DNA with primers Ipo323-hygroF and Ipo323-pKraR. Finally the hygromycin resistance marker gene *hph* was amplified from plasmid pCAMB-HPT-Hind (85) with the primer pair Hygro_MDR6_F/Hygro_ipo323_R or Hygro_MDR7_F/Hygro_ipo323_R, respectively. The three fragments (0.06 pmol each) were assembled with XhoI-EcoRI-digested pCAMB-HPT-Hind (0.02 pmol) using the Gibson Assembly Cloning kit (New England Biolabs, Evry, France) according to the supplier’s instructions. Half of the assembly reaction mixture was used to transform NEB 5-α competent *Escherichia coli* (New England Biolabs). The kanamycin-resistant colonies were PCR screened with the primers cited above. Positive clones were picked for plasmid extractions according to standard protocols (86). The extracted plasmids were checked again by EcoRI-NcoI restriction.

The resulting plasmids, pCAMBIA-MFS1(MDR6) and pCAMBIA-MFS1(MDR7), respectively, were introduced into *Agrobacterium tumefaciens* AGL1 competent cells by heat shock. Transformants were selected and isolated on LB broth with rifampin (20 µg ml^−1^), kanamycin (50 µg ml^−1^), and ampicillin (50 µg ml^−1^).

### *Z. tritici* transformation and analysis.

The *Agrobacterium*-mediated transformation procedure was performed as described by Zwiers and de Waard (90) according to the modifications made by Kramer et al. (85) with minor changes. Transformants were selected and isolated on hygromycin (100 µg ml^−1^) containing YPD medium. Fifty transformants for each plasmid were picked and isolated twice on selective YPD medium. All purified transformants were tested by PCR: genomic DNA was extracted from cells harvested on solid YPD medium with the GenElute Plant Genomic DNA Miniprep kit (Sigma-Aldrich). Primers Z4_110044_FW and Z4_110044_RV (Table S2) were used to PCR amplify the *MFS1* promoter in order to distinguish transformants devoid of the insert (700-bp amplicon) from those with the insert (1,200-bp amplicon) and from ectopic integrations (700 bp with an additional amplicon). Sixteen and 10 transformants, respectively, out of 50 (20 to 32%) had the *MFS1*^*MDR*^ allele integrated at the *MFS1* locus of IPO323.

### Fungicide sensitivity assays of selected transformants.

All validated transformants were tested for their sensitivity to various fungicides on solid YPD medium supplemented with tolnaftate (2 µg ml^−1^), terbinafine (0.015 µg ml^−1^), prochloraz (0.05 µg ml^−1^), metconazole (0.02 µg ml^−1^), boscalid (2 µg ml^−1^), bixafen (0.5 µg ml^−1^), and azoxystrobin (20 µg ml^−1^). All fungicides were supplied as 1,000× concentrated ethanol solutions. Strains were precultured in 5 ml of liquid YPD medium for 3 days at 18°C and 140 rpm. After measurement of the optical density at 600 nm (OD_600_), all cultures were adjusted with fresh YPD medium to the lowest measured OD. Three sequential 10-fold dilutions were prepared from all adjusted precultures. Three microliters of precultures and dilutions were spotted on each fungicide assay and control plate and incubated at 17°C in the dark for 5 days.
