# Correction for Omrane et al., “Plasticity of the *MFS1* Promoter Leads to Multidrug Resistance in the Wheat Pathogen *Zymoseptoria tritici*”

**DOI:** 10.1128/mSphere.00322-18

**Published:** 2018-06-27

**Authors:** Selim Omrane, Colette Audéon, Amandine Ignace, Clémentine Duplaix, Lamia Aouini, Gert Kema, Anne-Sophie Walker, Sabine Fillinger

**Affiliations:** a UMR BIOGER, INRA, AgroParisTech, Université Paris-Saclay, Thiverval-Grignon, France; b Wageningen University, Plant Research International, Wageningen, The Netherlands

## AUTHOR CORRECTION

Volume 2, no. 5, e00393-17, 2017, https://doi.org/10.1128/mSphere.00393-17. On page 10 of the PDF, Fig. 7B is incomplete, as it lists the insertion sequences of type IIb inserts only. To be complete, the legend should include the following statement: “The sequences of type II insertion sites are GGTAA/TTGTT (left) and TAAGG/TAGGT (right) for insert IIa and TAAGG/TAGTT (left) and TAAGG/TAGGT (right) for insert IIb.”

On page 13 of the PDF, in Table 4, the *MFS1* genotype of strain 12-VM-5E was incorrectly stated. It should say “Type II” rather than “Type I.”

On page 16 of the PDF, in the second sentence of Acknowledgments, the statement “Part of the study was financed by Ministère de l’Environnement, du Développement Durable et de l’Écologie (MEDDE) through the Ecophyto II program” should be replaced with “Part of this study had the financial support of the French Biodiversity Agency on Resistance and Pesticides call for research, with the fees for diffuse pollution coming from the Ecophyto plan, an action led by the Ministry for Agriculture and Food and the Ministry for Ecological and Solidary Transition.”

