# Supplementary material for: Plasticity of the MFS1 Promoter Leads to Multidrug Resistance in the Wheat Pathogen Zymoseptoria tritici
Source: mSphere. 2017 Oct 25;2(5):e00393-17. doi: 10.1128/mSphere.00393-17 (PMC5656749; doi:10.1128/mSphere.00393-17)
Supplement: TABLE S2 [file sph005172387st3.docx]

Table S2: Primers used in this study

| Primer ID ^a^ | Sequence (5’-3’)* | Purpose ^b^ |
| --- | --- | --- |
| MFS1_2F | GCAAGGATTCGGACTTGACG | Detection of *MFS1* promoter inserts (progeny, field-strains) |
| MFS1_4R | CTGCCGGTATCGTCGATGAC | Detection of *MFS1* promoter inserts (progeny, field-strains) |
| Z4_110044_FW | ACATGATCCCTGATCCGTTC | Analysis of *MFS1* promoter (transformants) |
| Z4_110044_RV | CGGCGACTTCTTGCTGAA | Analysis of *MFS1* promoter (transformants) |
| NFX1_ 11422FW | CACAATAGCATCGAGCAGGA | Progeny genotyping |
| NFX1_ 11422RV | GAGATCTGCGGTGCTTTGAT | Progeny genotyping |
| PYC_5UTR_FW | CCTTCTGGGTGGTCGACAT | Progeny genotyping |
| PYC_5UTR_RV | CCTCCCGCTCGAGTAAAACT | Progeny genotyping |
| MDR_pKr_F | gcttatcgatgggccccccctcgagAAAGGTGGCATCCGATGTAG | *MFS1* replacement construct |
| MDR6_hyg_R | tccagccaagTCTACTGCGGTTGGGAAATC | *MFS1* replacement construct |
| MDR7_hyg R | tccagccaagTGATGCAAGACAAGGAGC | *MFS1* replacement construct |
| Ipo323_hyg_F | atgccgaccgCACTGCCGATCATAGAGC | *MFS1* replacement construct |
| Ipo323_pKr_R | atgttgggcccggcgcgccgaattcTTTGAGTGCGATGAAGATG | *MFS1* replacement construct |
| Hygro_MDR6_F | ccgcagtagaCTTGGCTGGAGCTAGTGG | *MFS1* replacement construct |
| Hygro_MDR7_F | tcttgcatcaCTTGGCTGGAGCTAGTGG | *MFS1* replacement construct |
| Hygro_ipo323_R | atcggcagtgCGGTCGGCATCTACTCTATTC | *MFS1* replacement construct |
| *EF1α Fw*  *EF1α Rv*  *β-tubuline Fw*  *β-tubuline Rv*  *UBC Fw*  *UBC RV* | AAGATTGGTGGTATCGGAACAG  GACTTGACTTCGGTGGTGAC  AACGAGGCTCTCTACGACATCTG  GGCGGAGACGAGGTGGTTG  GTCTGCGGACCACAATACC  CGACCTTTCCTTGCCTCTG | *MFS1* expression (housekeeping gene)  *MFS1* expression (housekeeping gene)  *MFS1* expression (housekeeping gene)  *MFS1* expression (housekeeping gene)  *MFS1* expression (housekeeping gene)  *MFS1* expression (housekeeping gene) |

* lower case letters correspond to linker sequences for cloning purposes
