# Supplementary material for: Plasticity of the MFS1 Promoter Leads to Multidrug Resistance in the Wheat Pathogen Zymoseptoria tritici
Source: mSphere. 2017 Oct 25;2(5):e00393-17. doi: 10.1128/mSphere.00393-17 (PMC5656749; doi:10.1128/mSphere.00393-17)
Supplement: FIG S1 [file sph005172387sf1.pdf]

Fig. S1 : Sequence alignment of *MFS1* promoter of *Z. tritici* field strains

```
gi|110265120|gb   GCCGCAAGGATTCGGACTTGACGACTTTTCGGTGGACAGGACAGATT------------------CCGGAC-GGGGTAGGAATGCGAGGAGAGGGTAA----------------------
12-VM-4J          --------------GACTTGACGACTTTTCGGTGGACAGGACAGATT-CGTCCGCCGGCACTTGCCCGGAC-GGGGTAGGANTGCGAGGAGAGGGTAA-TTGTTTGTTTGTTTGTTTGTT
12_VM_5A          -----------------------------------ACAGGACAGATT-CGTCCGGCGCAACTTGCCCGGAC-GGGGTAGGAATGCGAGGAGAGGGTAA-TTGTTTGTTTGTTTGTTTGTT
12_VM_5C          ---------------GACTGACGAC-TTTCGGTGGACAGGACAGATT-CGTCCGGCGCAACTTGCCCGGAC-GGGGTAGGAATGCGAGGAGAGGGTAA-TTGTTTGTTTGTTTGTTTGTT
14_STDO_28        ------------TTCGACTGACGACTTTTCGGTGGACAGGACAGATT-CGTCCGGCGCAACTTGCCCGGAC-GGGGTAGGAATGCGAGGAGAGGGTAA-TTGTTTGTTTGTTTGTTTGTT
12_VM_5F          ----------------GCTGACGAC-TTTCGTGGGACAGGACAGATT-CGTCCGGCGCAACTTGCCCGGAC-GGGGTAGGAATGCGAGGAGAGGGTAA-TTGTTTGTTTGTTTGTTTGTT
12_VM_5E          ----------------GCTGACGACTTTTCGGTGGACAGGACAGATT-CGTCCGGCGC-ACTTGCCCGGAC-GGGGTAGGAATGCGAGGAGAGGGTAA-TTGTTTGTTTGTTTGTTTGTT
12_AGC_13C        ----CAAGGAATTCGACTTGACGACTTTTCGGTGGACAGGACAGATT-CGTCCGGCGCAACTTGCCCGGAC-GGGGTAGGAATGCGAGGAGAA---------------------------
12_VM_4F          ----CAAGGAATTCGACTTGACGACTTTTCGGTGGACAGGACAGATT-CGTCCGGCGCAACTTGCCCGGAC-GGGGTAGGAATGCGAGGAGAA---------------------------
ST_5548           ----CAAGGATTCGGACTTGACGACTTTTCGGTGGACAGGACAGATT-CGTCCGGCGCAACTTGCCCGGAC-GGGGTAGGAATGCGAGGAGAA---------------------------
14_AQ-1           ---------------GACTTGACGACTTTTCGGTGGACAGGACAGATT-CGGACGGGGCAACTTGCCCGGAC-GGGGTAGGAATGCGAGGAGAGGGTAA----------------------
14_STIRLO_13      ----CAAGGAATTCGACTTGACGACTTTTCGGTGGACAGGACAGATTCCGGACGGGGCAACTTGCCCGGAC-GGGGTAGGAATGCGAGGAGAG--------------------------
15_PN_3           ----CAAGGATTCGGACTTGACGACTTTTCGGTGGACAGGACAGATT-------------------CGGAC-GGGGTAGGAATGCGAGGAGAGGGTAA----------------------
STDP_04915        ----CAAGGATTTCGACTTGACGACTTTTCGGTGGACAGGACAGATT-CGGACGGGGCAACTTGCCCGGAC-GGGGTAGGAATGCGAGGAGAGGGTAA----------------------
15_OU_1D          -------------GGACTTGACGACTTTTCGGTGGACAGGACAGATT-CGGACGGGGCAACTTGCCCGGAC-GGGGTAGGAATGCGAGGAGAG--------------------------
15-PQ-8B          ------------------------------------------------------------------------------------CGAGGAGAGGGTAA----------------------
15_PQ-2B          ---------------ACTTGACGACTTTTCGGTGGACAGGACAGATT-CGGACGGGGCAACTTGCCCGGAC-GGGGTAGGAATGCGAGGAGAGGGTAA----------------------
09-ASA-3a         GCCGCAAGGATTCGGACTTGACGACTTTTCGGTGGACAGGACAGATT-CGGACGGGGCAACTTGCCCGGAC-GGGGTAGGAATGCGAGGAGAGGGTAA----------------------
13-AHJ-8C         -----------------------------------------------------------------------------------GCGAGGAGAGGGTAA----------------------
14-STDK           ----------------------------------------------------------GACTTGCCCGGAC-GGGGTAGG-ATGCGAGGAGAGGGTAA----------------------
10-BNE-35         -----------------------------------------AGAGAG-ATCGGCGGTGCACTTGCCCGGAC-GGGGTAGG-ATGCGAGGAGAGGGTAA----------------------
15-PQ-6A          -----------------------------------------AGAGAG-ATCGGCGGTGCACTTGCCCGGAC-GGGGTAGGAATGCGAGGAGAGGGTAA----------------------
09-ASA-10b.pz     ------------------------------------------GAGAG-ATCGGAGGGGCACTTGCCCGGAC-GGGGTAGGAATGCGAGGAGAGGGTAA----------------------
12-VM-7A          ------------------------------------------GAGAG-ATCGGAGGTGCACTTGCCCGGAC-GGGGTAGGAATGCGAGGAGAGGGTAA----------------------
14-EG-A1          ------------------------------------------GAGAG-ATCGGAGGGGCACTTGCCCGGAC-GGGGTAGGAATGCGAGGAGAGGGTAA----------------------
09-CB1            GCCGCAAGGATTCGGACTTGACGACTTTTCGGTGGACAGGACAGATT-CGGACGGGGCAACTTGCCCGGAC-GGGGTAGGAATGCGAGGAGAGGGTAA----------------------
14-AK1b           ---------------ACTTGACGACTTTTCGGTGGACAGGACAGATT------------------CCGGAC-GGGGTAGGAATGCGAGGAGAGGGTAAGGTAGTTGTTTGTTTGTTTGTT
consensus                        ................................ ..  ... ............... ............********......
                  .......370.......380.......390.......400.......410.......420.......430.......440.......450.......460.......470.......480

gi|110265120|gb   ------------------------------------------------------------------------------------------------------------------------
12-VM-4J          TGTTTGTTTGTTCCATTTACGTCCTGTAGGAGTCTATGACTATGTAGGACGGCCTTGC-----AAGCAAGGCAGTAGATCTATTTACAATCAAATTCTTTCAGTATTCTTTGACCTTAGG
12_VM_5A          TGTTTGTTTGTTCCATTTACGTCCTGTAGGAGTCTATGACTATGTAGGACGGCCTTGC-----AAGCAAGGCAGTAGATCTATTTACAATCAAATTCTTTCAGTATTCTTTGACCTTAGG
12_VM_5C          TGTTTGTTTGTTCCATTTACGTCCTGTAGGAGTCTATGACTATGTAGGACGGCCTTGC-----AAGCAAGGCAGTAGATCTATTTACAATCAAATTCTTTCAGTATTCTTTGACCTTAGG
14_STDO_28        TGTTTGTTTGTTCCATTTACGTCCTGTAGGAGTCTATGACTATGTAGGACGGCCTTGC-----AAGCAAGGCAGTAGATCTATTTACAATCAAATTCTTTCAGTATTCTTTGACCTTAGG
12_VM_5F          TGTTTGTTTGTTCCATTTACGTCCTGTAGGAGTCTATGACTATGTAGGACGGCCTTGC-----AAGCAAGGCAGTAGATCTATTTACAATCAAATTCTTTCAGTATTCTTTGACCTTAGG
12_VM_5E          TGTTTGTTTGTTCCATTTACGTCCTGTAGGAGTCTATGACTATGTAGGACGGCCTTGC-----AAGCAAGGCAGTAGATCTATTTACAATCAAATTCTTTCAGTATTCTTTGACCTTAGG
12_AGC_13C        ------------------------------------------------------------------------GGTAGAAC----------------------------------------
12_VM_4F          ------------------------------------------------------------------------GGTAGAAC----------------------------------------
ST_5548           ------------------------------------------------------------------------GGTAGAAC----------------------------------------
14_AQ-1           ------------------------------------------------------------------------GGTAGAAC----------------------------------------
14_STIRLO_13      ------------------------------------------------------------------------GGTAGAAC----------------------------------------
15_PN_3           ------------------------------------------------------------------------GGTAGAAC----------------------------------------
STDP_04915        ------------------------------------------------------------------------GGTAGAAC----------------------------------------
15_OU_1D          ------------------------------------------------------------------------GGTAGAAC----------------------------------------
15-PQ-8B          ------------------------------------------------------------------------------------------------------------------------
15_PQ-2B          ------------------------------------------------------------------------------------------------------------------------
09-ASA-3a         ------------------------------------------------------------------------------------------------------------------------
13-AHJ-8C         ------------------------------------------------------------------------------------------------------------------------
14-STDK           ------------------------------------------------------------------------------------------------------------------------
10-BNE-35         ------------------------------------------------------------------------------------------------------------------------
15-PQ-6A          ------------------------------------------------------------------------------------------------------------------------
09-ASA-10b.pz     ------------------------------------------------------------------------------------------------------------------------
12-VM-7A          ------------------------------------------------------------------------------------------------------------------------
14-EG-A1          ------------------------------------------------------------------------------------------------------------------------
09-CB1            ------------------------------------------------------------------------------------------------------------------------
14-AK1b           TGTTTGTTTGTTCCATTTACGTCCTTTTGGAGTCTAAGACTATGTAGGACGGCTTTGCCCTAAAGGCAAAGCAGTAGATCTAGTTACAATCAGATTCTTT-AGTATTCTTTGACCTTAGG
consensus                                                                                 ...... .
                  .......490.......500.......510.......520.......530.......540.......550.......560.......570.......580.......590.......600

gi|110265120|gb   ------------------------------------------------------------------------------------------------------------------------
12-VM-4J          GGAAACCCCGTGCCTAAGGCAGTGCCAAAGGACTTTAAACAAATGCAAGACAAAGGAAATCGAACGAGTGTCGTTGTCTGTTGTCTGCAGCTGGGGCTGCAGAATTTTCGTTGTCTGCAG
```

```
12_VM_5A        GGAAACCCCGTGCCTAAGGCAGTGCCAAAGGACTTTAAACAAATGCAAGACAAAGGAAATCGAACGAGTGTCGTTGTCTGTTGTCTGCAGCTGGGGCTGCAGAATTTTCGTTGTCTGCAG
12_VM_5C        GGAAACCCCGTGCCTAAGGCAGTGCCAAAGGACTTTAAACAAATGCAAGACAAAGGAAATCGAACGAGTGTCGTTGTCTGTTGTCTGCAGCTGGGGCTGCAGAATTTTCGTTGTCTGCAG
14_STDO_28      GGAAACCCCGTGCCTAAGGCAGTGCCAAAGGACTTTAAACAAATGCAAGACAAAGGAAATCGAACGAGTGTCGTTGTCTGTTGTCTGCAGCTGGGGCTGCAGAATTTTCGTTGTCTGCAG
12_VM_5F        GGAAACCCCGTGCCTAAGGCAGTGCCAAAGGACTTTAAACAAATGCAAGACAAAGGAAATCGAACGAGTGTCGTTGTCTGTTGTCTGCAGCTGGGGCTGCAGAATTTTCGTTGTCTGCAG
12_VM_5E        GGAAACCCCGTGCCTAAGGCAGTGCCAAAGGACTTTAAACAAATGCAAGACAAAGGAAATCGAACGAGTGTCGTTGTCTGTTGTCTGCAGCTGGGGCTGCAGAATTTTCGTTGTCTGCAG
12_AGC_13C      ------------------------------------------------------------------------------------------------------------------------
12_VM_4F        ------------------------------------------------------------------------------------------------------------------------
ST_5548         ------------------------------------------------------------------------------------------------------------------------
14_AQ-1         ------------------------------------------------------------------------------------------------------------------------
14_STIRLO_13    ------------------------------------------------------------------------------------------------------------------------
15_PN_3         ------------------------------------------------------------------------------------------------------------------------
STDP_04915      ------------------------------------------------------------------------------------------------------------------------
15_OU_1D        ------------------------------------------------------------------------------------------------------------------------
15-PQ-8B        ------------------------------------------------------------------------------------------------------------------------
15_PQ-2B        ------------------------------------------------------------------------------------------------------------------------
09-ASA-3a       ------------------------------------------------------------------------------------------------------------------------
13-AHJ-8C       ------------------------------------------------------------------------------------------------------------------------
14-STDK         ------------------------------------------------------------------------------------------------------------------------
10-BNE-35       ------------------------------------------------------------------------------------------------------------------------
15-PQ-6A        ------------------------------------------------------------------------------------------------------------------------
09-ASA-10b.pz   ------------------------------------------------------------------------------------------------------------------------
12-VM-7A        ------------------------------------------------------------------------------------------------------------------------
14-EG-A1        ------------------------------------------------------------------------------------------------------------------------
09-CB1          ------------------------------------------------------------------------------------------------------------------------
14-AK1b         GGAGACCCCGTGCCTAAGGCAATGCCAAAGGACTTCAAAACAGCGCAAGACAAAGGAAATCAAACGAGTGTCGTTGTCTGCAGCCGG-----GGCTGCAGAATTTTCGCGCCAAGCTTTT
consensus
                .......610.......620.......630.......640.......650.......660.......670.......680.......690.......700.......710.......720

gi|110265120|gb -----------------------------------------------------------------------------------------------------------------GGTAGGT
12-VM-4J        CTGGGGCTGCAGAATTTTCGTTGTCTGCAGCTAAGGCTGCAGAATTTTTTTGCGCCAAGCTTTTTGCAGAATTTTCAGCGGATCCTTCCGCTTCGAGGAGAGGGTAATAATAAGGTAGGT
12_VM_5A        CTGGGGCTGCAGAATTTTCGTTGTCTGCAGCTAAGGCTGCAGAATTTTTTTGCGCCAAGCTTTTTGCAGAATTTTCAGCGGATCCTTCCGCTTCGAGGAGAGGGTAATAATAAGGTAGGT
12_VM_5C        CTGGGGCTGCAGAATTTTCGTTGTCTGCAGCTAAGGCTGCAGAATTTTTTTGCGCCAAGCTTTTTGCAGAATTTTCAGCGGATCCTTCCGCTTCGAGGAGAGGGTAATAATAAGGTAGGT
14_STDO_28      CTGGGGCTGCAGAATTTTCGTTGTCTGCAGCTAAGGCTGCAGAATTTTTTTGCGCCAAGCTTTTTGCAGAATTTTCAGCGGATCCTTCCGCTTCGAGGAGAGGGTAATAATAAGGTAGGT
12_VM_5F        CTGGGGCTGCAGAATTTTCGTTGTCTGCAGCTAAGGCTGCAGAATTTTTTTGCGCCAAGCTTTTTGCAGAATTTTCAGCGGATCCTTCCGCTTCGAGGAGAGGGTAATAATAAGGTAGGT
12_VM_5E        CTGGGGCTGCAGAATTTTCGTTGTCTGCAGCTAAGGCTGCAGAATTTTTTTGCGCCAAGCTTTTTGCAGAATTTTCAGCGGATCCTTCCGCTTCGAGGAGAGGGTAATAATAAGGTAGGT
12_AGC_13C      -------------------------------------------------------------------------------------------------------------------TGGGT
12_VM_4F        -------------------------------------------------------------------------------------------------------------------TGGGT
ST_5548         -------------------------------------------------------------------------------------------------------------------TGGGT
14_AQ-1         -------------------------------------------------------------------------------------------------------------------TAGGT
14_STIRLO_13    -------------------------------------------------------------------------------------------------------------------TGGGT
15_PN_3         -------------------------------------------------------------------------------------------------------------------TAGGT
STDP_04915      -------------------------------------------------------------------------------------------------------------------TAGGT
15_OU_1D        -------------------------------------------------------------------------------------------------------------------TGGGT
15-PQ-8B        -----------------------------------------------------------------------------------------------------------------GGTAGGT
15_PQ-2B        -----------------------------------------------------------------------------------------------------------------GGTAGGT
09-ASA-3a       -----------------------------------------------------------------------------------------------------------------GGTAGGT
13-AHJ-8C       -----------------------------------------------------------------------------------------------------------------GGTAGGT
14-STDK         -----------------------------------------------------------------------------------------------------------------GGTAGGT
10-BNE-35       -----------------------------------------------------------------------------------------------------------------GGTAGGT
15-PQ-6A        -----------------------------------------------------------------------------------------------------------------GGTAGGT
09-ASA-10b.pz   -----------------------------------------------------------------------------------------------------------------GGTAGGT
12-VM-7A        -----------------------------------------------------------------------------------------------------------------GGTAGGT
14-EG-A1        -----------------------------------------------------------------------------------------------------------------GGTAGGT
09-CB1          -----------------------------------------------------------------------------------------------------------------GGTAGGT
14-AK1b         GCAGAATTTTCAGAGTGTCGTTGTCTGCAGCCCAAGGCTGCAGAATTTTCGCGCCAAGCTTTTTGCAGAATTTTCAGGGTAA-------------------------------GGTAGGT
consensus                                                                                                                        ..*.***
                .......730.......740.......750.......760.......770.......780.......790.......800.......810.......820.......830.......840
```

```
gi|110265120|gb  GAACACCTTATACTCCGTTTCTTTCCCATTCCTCTCCTCCATCATCACAGTCATCAGAT-------------------------------------------------------------
12-VM-4J         GAACACCTTATACTCCGTTTCTTTCCCATTCCTCTCCTCCATCATCACAGTCATCAGAT-------------------------------------------------------------
12_VM_5A         GAACACCTTATACTCCGTTTCTTTCCCATTCCTCTCCTCCATCATCACAGTCATCAGAT-------------------------------------------------------------
12_VM_5C         GAACACCTTATACTCCGTTTCTTTCCCATTCCTCTCCTCCATCATCACAGTCATCAGAT-------------------------------------------------------------
14_STDO_28       GAACACCTTATACTCCGTTTCTTTCCCATTCCTCTCCTCCATCATCACAGTCATCAGAT-------------------------------------------------------------
12_VM_5F         GAACACCTTATACTCCGTTTCTTTCCCATTCCTCTCCTCCATCATCACAGTCATCAGAT-------------------------------------------------------------
12_VM_5E         GAACACCTTATACTCCGTTTCTTTCCCATTCCTCTCCTCCATCATCACAGTCATCAGAT-------------------------------------------------------------
12_AGC_13C       GAA-----TATACTCCGATTCTTTCCC-TTTCTCTCCTTCATCATCACAGTCATCAGAT-------------------------------------------------------------
12_VM_4F         GAA-----TATACTCCGATTCTTTCCC-TTTCTCTCCTTCATCATCACAGTCATCAGAT-------------------------------------------------------------
ST_5548          GAA-----TATACTCCGATTCTTTCCC-TTTCTCTCCTTCATCATCACAGTCATCAGAT-------------------------------------------------------------
14_AQ-1          GAACACCTTGTACTCCGTTTCTTTCCCATTCCTCTCCTCCATCATCACAGTCATCAGAT-------------------------------------------------------------
14_STIRLO_13     GAA-----TATACTCCGATTCTTTCCCATTCCTCTCCTCCATCATCACAGTCATCAGAT-------------------------------------------------------------
15_PN_3          GAACACCTTATACTCCGATTCTTTCCCATTCCTCTCCTCCATCATCACAGTCATCAGAT-------------------------------------------------------------
STDP_04915       GAACACCTTGTACTCCGTTTCTTTCCCATTCCTCTCCTCCATCATCACAGTCATCAGAT-------------------------------------------------------------
15_OU_1D         GAA-----TATACTCCGATTCTTTCCC-TTTCTCTCCTTCATCATCACAGTCATCAGAT-------------------------------------------------------------
15-PQ-8B         GAACACCTTATACTCCGTTTCTTTCCCATTCCTCTCCTCCATCATCACAGTCATCAGAT-------------------------------------------------------------
15_PQ-2B         GAACACCTTATACTCCGTTTCTTTCCCATTCCTCTCCTCCATCATCACAGTCATCAGAT-------------------------------------------------------------
09-ASA-3a        GAACACCTTATACTCCGATTCTTTCCC-TTTCTCTCCTTCATCATCACAGTCATCAGATCCGCATGTTGAAAATAAGCTGAATTCCGGAAGGCGTTGGGCGTTGGTGCCGTTTTCGGACT
13-AHJ-8C        GAACACCTTATACTCCGATTCTTTCCC-TTTCTCTCCTTCATCATCACAGTCATCAGATCCGCATGTTGAAAATAAGCTGAATTCCGGAAGGCGTTGGGCGTTGGTGCCGTTTTCGGACT
14-STDK          GAACACCTTATACTCCGATTCTTTCCC-TTTCTCTCCTTCATCATCACAGTCATCAGATCCGCATGTTGAAAATAAGCTGAATTCCGGAAGGCGTTGGGCGTTGGTGCCGTTTTCGGACT
10-BNE-35        GAACACCTTATACTCCGATTCTTTCCC-TTTCTCTCCTTCATCATCACAGTCATCAGATCCGCATGTTGAAAATAAGCTGAATTCCGGAAGGCGTTGGGCGTTGGTGCCGTTTTCGGACT
15-PQ-6A         GAACACCTTATACTCCGATTCTTTCCC-TTTCTCTCCTTCATCATCACAGTCATCAGATCCGCATGTTGAAAATAAGCTGAATTCCGGAAGGCGTTGGGCGTTGGTGCCGTTTTCGGACT
09-ASA-10b.pz    GAACACCTTATACTCCGATTCTTTCCC-TTTCTCTCCTTCATCATCACAGTCATCAGATCCGCATGTTGAAAATAAGCTGAATTCCGGAAGGCGTTGGGCGTTGGTGCCGTTTTCGGACT
12-VM-7A         GAACACCTTATACTCCGATTCTTTCCC-TTTCTCTCCTTCATCATCACAGTCATCAGATCCGCATGTTGAAAATAAGCTGAATTCCGGAAGGCGTTGGGCGTTGGTGCCGTTTTCGGACT
14-EG-A1         GAACACCTTATACTCCGATTCTTTCCC-TTTCTCTCCTTCATCATCACAGTCATCAGATCCGCATGTTGAAAATAAGCTGAATTCCGGAAGGCGTTGGGCGTTGGTGCCGTTTTCGGACT
09-CB1           GAACACCTTATACTCCGATTCTTTCCC-TTTCTCTCCTTCATCATCACAGTCATCAGATCCGCATGTTGAAAATAAGCTGAATTCCGGAAGGCGTTGGGCGTTGGTGCCGTTTTCGGACT
14-AK1b          GAACACCTTATACTCCGTTTCTTTCCC-TTTCTCTCCTCCATCATCACAGTCATCAGAT-------------------------------------------------------------
consensus        ***.....*.*******.********* **.*******.********************
                 .......850.......860.......870.......880.......890.......900.......910.......920.......930.......940.......950.......960

gi|110265120|gb  ------------------------------------------------------------------------------------------------------------------------
12-VM-4J         ------------------------------------------------------------------------------------------------------------------------
12_VM_5A         ------------------------------------------------------------------------------------------------------------------------
12_VM_5C         ------------------------------------------------------------------------------------------------------------------------
14_STDO_28       ------------------------------------------------------------------------------------------------------------------------
12_VM_5F         ------------------------------------------------------------------------------------------------------------------------
12_VM_5E         ------------------------------------------------------------------------------------------------------------------------
12_AGC_13C       ------------------------------------------------------------------------------------------------------------------------
12_VM_4F         ------------------------------------------------------------------------------------------------------------------------
ST_5548          ------------------------------------------------------------------------------------------------------------------------
14_AQ-1          ------------------------------------------------------------------------------------------------------------------------
14_STIRLO_13     ------------------------------------------------------------------------------------------------------------------------
15_PN_3          ------------------------------------------------------------------------------------------------------------------------
STDP_04915       ------------------------------------------------------------------------------------------------------------------------
15_OU_1D         ------------------------------------------------------------------------------------------------------------------------
15-PQ-8B         ------------------------------------------------------------------------------------------------------------------------
15_PQ-2B         ------------------------------------------------------------------------------------------------------------------------
09-ASA-3a        AGTGGATTTCTGAACCGGCGTTGGTCGACGATCTTTTACCCGATTAGGACTAGCGCGGAATCGTACCGGCGTTGGTCGAAGATTCCAGACGCACCTTACCCGATTCGGACTAGCGGTTAC
13-AHJ-8C        AGTGGATTTCTGAACCGGCGTTGGTCGACGATCTTTTACCCGATTAGGACTAGCGCGGAATCGTACCGGCGTTGGTCGAAGATTCCAGACGCACCTTACCCGATTCGGACTAGCGGTTAC
14-STDK          AGTGGATTTCTGAACCGGCGTTGGTCGACGATCTTTTACCCGATTAGGACTAGCGCGGAATCGTACCGGCGTTGGTCGAAGATTCCAGACGCACCTTACCCGATTCGGACTAGCGGTTAC
10-BNE-35        AGTGGATTTCTGAACCGGCGTTGGTCGACGATCTTTTACCCGATTAGGACTAGCGCGGAATCGTACCGGCGTTGGTCGAAGATTCCAGACGCACCTTACCCGATTCGGACTAGCGGTTAC
15-PQ-6A         AGTGGATTTCTGAACCGGCGTTGGTCGACGATCTTTTACCCGATTAGGACTAGCGCGGAATCGTACCGGCGTTGGTCGAAGATTCCAGACGCACCTTACCCGATTCGGACTAGCGGTTAC
09-ASA-10b.pz    AGTGGATTTCTGAACCGGCGTTGGTCGACGATCTTTTACCCGATTAGGACTAGCGCGGAATCGTACCGGCGTTGGTCGAAGATTCCAGACGCACCTTACCCGATTCGGACTAGCGGTTAC
12-VM-7A         AGTGGATTTCTGAACCGGCGTTGGTCGACGATCTTTTACCCGATTAGGACTAGCGCGGAATCGTACCGGCGTTGGTCGAAGATTCCAGACGCACCTTACCCGATTCGGACTAGCGGTTAC
14-EG-A1         AGTGGATTTCTGAACCGGCGTTGGTCGACGATCTTTTACCCGATTAGGACTAGCGCGGAATCGTACCGGCGTTGGTCGAAGATTCCAGACGCACCTTACCCGATTCGGACTAGCGGTTAC
09-CB1           AGTGGATTTCTGAACCGGCGTTGGTCGACGATCTTTTACCCGATTAGGACTAGCGCGGAATCGTACCGGCGTTGGTCGAAGATTCCAGACGCACCTTACCCGATTCGGACTAGCGGTTAC
14-AK1b          ------------------------------------------------------------------------------------------------------------------------
consensus
                 .......970.......980.......990......1000......1010......1020......1030......1040......1050......1060......1070......1080
```

```
gi|110265120|gb ------------------------------------------------------------------------------------------------------------------------
12-VM-4J        ------------------------------------------------------------------------------------------------------------------------
12_VM_5A        ------------------------------------------------------------------------------------------------------------------------
12_VM_5C        ------------------------------------------------------------------------------------------------------------------------
14_STDO_28      ------------------------------------------------------------------------------------------------------------------------
12_VM_5F        ------------------------------------------------------------------------------------------------------------------------
12_VM_5E        ------------------------------------------------------------------------------------------------------------------------
12_AGC_13C      ------------------------------------------------------------------------------------------------------------------------
12_VM_4F        ------------------------------------------------------------------------------------------------------------------------
ST_5548         ------------------------------------------------------------------------------------------------------------------------
14_AQ-1         ------------------------------------------------------------------------------------------------------------------------
14_STIRLO_13    ------------------------------------------------------------------------------------------------------------------------
15_PN_3         ------------------------------------------------------------------------------------------------------------------------
STDP_04915      ------------------------------------------------------------------------------------------------------------------------
15_OU_1D        ------------------------------------------------------------------------------------------------------------------------
15-PQ-8B        ------------------------------------------------------------------------------------------------------------------------
15_PQ-2B        ------------------------------------------------------------------------------------------------------------------------
09-ASA-3a       CCGATTGGGACTAGCTCATGTCTAGCTCGTCCCAATCGGGACTAGCTCAAAATCGTACCGGCGTTGGTCGAAAATTCTAGAAACTCTTCTTCTTCGAATTGTTTCTTTATCTGTCTACTG
13-AHJ-8C       CCGATTGGGACTAGCTCATGTCTAGCTCGTCCCAATCGGGACTAGCTCAAAATCGTACCGGCGTTGGTCGAAAATTCTAGAAACTCTTCTTCTTCGAATTGTTTCTTTATCTGTCTACTG
14-STDK         CCGATTGGGACTAGCTCATGTCTAGCTCGTCCCAATCGGGACTAGCTCAAAATCGTACCGGCGTTGGTCGAAAATTCTAGAAACTCTTCTTCTTCGAATTGTTTCTTTATCTGTCTACTG
10-BNE-35       CCGATTGGGACTAGCTCATGTCTAGCTCGTCCCAATCGGGACTAGCTCAAAATCGTACCGGCGTTGGTCGAAAATTCTAGAAACTCTTCTTCTTCGAATTGTTTCTTTATCTGTCTACTG
15-PQ-6A        CCGATTGGGACTAGCTCATGTCTAGCTCGTCCCAATCGGGACTAGCTCAAAATCGTACCGGCGTTGGTCGAAAATTCTAGAAACTCTTCTTCTTCGAATTGTTTCTTTATCTGTCTACTG
09-ASA-10b.pz   CCGATTGGGACTAGCTCATGTCTAGCTCGTCCCAATCGGGACTAGCTCAAAATCGTACCGGCGTTGGTCGAAAATTCTAGAAACTCTTCTTCTTCGAATTGTTTCTTTATCTGTCTACTG
12-VM-7A        CCGATTGGGACTAGCTCATGTCTAGCTCGTCCCAATCGGGACTAGCTCAAAATCGTACCGGCGTTGGTCGAAAATTCTAGAAACTCTTCTTCTTCGAATTGTTTCTTTATCTGTCTACTG
14-EG-A1        CCGATTGGGACTAGCTCATGTCTAGCTCGTCCCAATCGGGACTAGCTCAAAATCGTACCGGCGTTGGTCGAAAATTCTAGAAACTCTTCTTCTTCGAATTGTTTCTTTATCTGTCTACTG
09-CB1          CCGATTGGGACTAGCTCATGTCTAGCTCGTCCCAATCGGGACTAGCTCAAAATCGTACCGGCGTTGGTCGAAAATTCTAGAAACTCTTCTTCTTCGAATTGTTTCTTTATCTGTCTACTG
14-AK1b         ------------------------------------------------------------------------------------------------------------------------
consensus
                ......1090......1100......1110......1120......1130......1140......1150......1160......1170......1180......1190......1200

gi|110265120|gb ------------------------------------------------------------------------------------------------------------------------
12-VM-4J        ------------------------------------------------------------------------------------------------------------------------
12_VM_5A        ------------------------------------------------------------------------------------------------------------------------
12_VM_5C        ------------------------------------------------------------------------------------------------------------------------
14_STDO_28      ------------------------------------------------------------------------------------------------------------------------
12_VM_5F        ------------------------------------------------------------------------------------------------------------------------
12_VM_5E        ------------------------------------------------------------------------------------------------------------------------
12_AGC_13C      ------------------------------------------------------------------------------------------------------------------------
12_VM_4F        ------------------------------------------------------------------------------------------------------------------------
ST_5548         ------------------------------------------------------------------------------------------------------------------------
14_AQ-1         ------------------------------------------------------------------------------------------------------------------------
14_STIRLO_13    ------------------------------------------------------------------------------------------------------------------------
15_PN_3         ------------------------------------------------------------------------------------------------------------------------
STDP_04915      ------------------------------------------------------------------------------------------------------------------------
15_OU_1D        ------------------------------------------------------------------------------------------------------------------------
15-PQ-8B        ------------------------------------------------------------------------------------------------------------------------
15_PQ-2B        ------------------------------------------------------------------------------------------------------------------------
09-ASA-3a       GTTTCGACGATTACGTCATCGATTACGCGTCCTACGTCATCGATTACGCGTCCTACGTCATAGATTACGCGTCTAATCGACGTATACGCGTATACAGAAGCTTCTAGATAGCTAGTAAAG
13-AHJ-8C       GTTTCGACGATTACGTCATCGATTACGCGTCCTACGTCATCGATTACGCGTCCTACGTCATAGATTACGCGTCTAATCGACGTATACGCGTATACAGAAGCTTCTAGATAGCTAGTAAAG
14-STDK         GTTTCGACGATTACGTCATCGATTACGCGTCCTACGTCATCGATTACGCGTCCTACGTCATAGATTACGCGTCTAATCGACGTATACGCGTATACAGAAGCTTCTAGATAGCTAGTAAAG
10-BNE-35       GTTTCGACGATTACGTCATCGATTACGCGTCCTACGTCATCGATTACGCGTCCTACGTCATAGATTACGCGTCTAATCGACGTATACGCGTATACAGAAGCTTCTAGATAGCTAGTAAAG
15-PQ-6A        GTTTCGACGATTACGTCATCGATTACGCGTCCTACGTCATCGATTACGCGTCCTACGTCATAGATTACGCGTCTAATCGACGTATACGCGTATACAGAAGCTTCTAGATAGCTAGTAAAG
09-ASA-10b.pz   GTTTCGACGATTACGTCATCGATTACGCGTCCTACGTCATCGATTACGCGTCCTACGTCATAGATTACGCGTCTAATCGACGTATACGCGTATACAGAAGCTTCTAGATAGCTAGTAAAG
12-VM-7A        GTTTCGACGATTACGTCATCGATTACGCGTCCTACGTCATCGATTACGCGTCCTACGTCATAGATTACGCGTCTAATCGACGTATACGCGTATACAGAAGCTTCTAGATAGCTAGTAAAG
14-EG-A1        GTTTCGACGATTACGTCATCGATTACGCGTCCTACGTCATCGATTACGCGTCCTACGTCATAGATTACGCGTCTAATCGACGTATACGCGTATACAGAAGCTTCTAGATAGCTAGTAAAG
09-CB1          GTTTCGACGATTACGTCATCGATTACGCGTCCTACGTCATCGATTACGCGTCCTACGTCATAGATTACGCGTCTAATCGACGTATACGCGTATACAGAAGCTTCTAGATAGCTAGTAAAG
14-AK1b         ------------------------------------------------------------------------------------------------------------------------
consensus
                ......1210......1220......1230......1240......1250......1260......1270......1280......1290......1300......1310......1320
```

```
gi|110265120|gb --------------------------------------------------------------------------------------------------CCGCACAACAATCATTCGGGTT
12-VM-4J        --------------------------------------------------------------------------------------------------CCGCACAACAATCATTCGGGTT
12_VM_5A        --------------------------------------------------------------------------------------------------CCGCACAACAATCATTCGGGTT
12_VM_5C        --------------------------------------------------------------------------------------------------CCGCACAACAATCATTCGGGTT
14_STDO_28      --------------------------------------------------------------------------------------------------CCGCACAACAATCATTCGGGTT
12_VM_5F        --------------------------------------------------------------------------------------------------CCGCACAACAATCATTCGGGTT
12_VM_5E        --------------------------------------------------------------------------------------------------CCGCACAACAATCATTCGGGTT
12_AGC_13C      --------------------------------------------------------------------------------------------------CCGCACAACAATCATTCGGGTT
12_VM_4F        --------------------------------------------------------------------------------------------------CCGCACAACAATCA------TT
ST_5548         --------------------------------------------------------------------------------------------------CCGCACAACAATCATTCGGGTT
14_AQ-1         --------------------------------------------------------------------------------------------------CCGCACAACAATCATTCGGGTT
14_STIRLO_13    --------------------------------------------------------------------------------------------------CCGCACAACAATCATTCGGGTT
15_PN_3         --------------------------------------------------------------------------------------------------CCGCACAACAATCATTCGGGTT
STDP_04915      --------------------------------------------------------------------------------------------------CCGCACAACAATCATTCGGGTT
15_OU_1D        --------------------------------------------------------------------------------------------------CCGCACAACAATCA------TT
15-PQ-8B        --------------------------------------------------------------------------------------------------CCGCACAACAATCATTCGGGTT
15_PQ-2B        --------------------------------------------------------------------------------------------------CCGCACAACAATCATTCGGGTT
09-ASA-3a       GGCATATAAGGGTTAGAAACGTCCGTAGAATCGATAGCAAGCAATTGAACACTTTCTACCTCTTTTACCCCCTTGGCTATTTCTTCCACTTTTTCATACCGCACAACAATCA------TT
13-AHJ-8C       GGCATATAAGGGTTAGAAACGTCCGTAGAATCGATAGCAAGCAATTGAACACTTTCTACCTCTTTTACCCCCTTGGCTATTTCTTCCACTTTTTCATACCGCACAACAATCA------TT
14-STDK         GGCATATAAGGGTTAGAAACGTCCGTAGAATCGATAGCAAGCAATTGAACACTTTCTACCTCTTTTACCCCCTTGGCTATTTCTTCCACTTTTTCATACCGCACAACAATCA------TT
10-BNE-35       GGCATATAAGGGTTAGAAACGTCCGTAGAATCGATAGCAAGCAATTGAACACTTTCTACCTCTTTTACCCCCTTGGCTATTTCTTCCACTTTTTCATACCGCACAACAATCA------TT
15-PQ-6A        GGCATATAAGGGTTAGAAACGTCCGTAGAATCGATAGCAAGCAATTGAACACTTTCTACCTCTTTTACCCCCTTGGCTATTTCTTCCACTTTTTCATACCGCACAACAATCA------TT
09-ASA-10b.pz   GGCATATAAGGGTTAGAAACGTCCGTAGAATCGATAGCAAGCAATTGAACACTTTCTACCTCTTTTACCCCCTTGGCTATTTCTTCCACTTTTTCATACCGCACAACAATCA------TT
12-VM-7A        GGCATATAAGGGTTAGAAACGTCCGTAGAATCGATAGCAAGCAATTGAACACTTTCTACCTCTTTTACCCCCTTGGCTATTTCTTCCACTTTTTCATACCGCACAACAATCA------TT
14-EG-A1        GGCATATAAGGGTTAGAAACGTCCGTAGAATCGATAGCAAGCAATTGAACACTTTCTACCTCTTTTACCCCCTTGGCTATTTCTTCCACTTTTTCATACCGCACAACAATCA------TT
09-CB1          GGCATATAAGGGTTAGAAACGTCCGTAGAATCGATAGCAAGCAATTGAACACTTTCTACCTCTTTTACCCCCTTGGCTATTTCTTCCACTTTTTCATACCGCACAACAATCA------TT
14-AK1b         --------------------------------------------------------------------------------------------------CCGCACAACAATCA------TT
consensus                                                                                                           **************......**
                ......1330......1340......1350......1360......1370......1380......1390......1400......1410......1420......1430......1440

gi|110265120|gb CGGTTCCTTCCCGTCATACTCATTGCCATTGGAGCTGAAAGGTCGAATTGCTGCTGCCTTGGGTCTCGCTTCGCTTCTGTGATTC-GACTTGTTCCGCCGTTGGATCTGGTGGAACGGAG
12-VM-4J        CGGTTCCTTCCCGTCATACTCATTGCCATTGGAGCTGAAAGGTCGAATTGCTGCTGCCTTGGGTCTCGCTTCGCTTCTGTGATTC-GACTTGTTCCGCCGTTGGATCTGGTGGAACGGAG
12_VM_5A        CGGTTCCTTCCCGTCATACTCATTGCCATTGGAGCTGAAAGGTCGAATTGCTGCTGCCTTGGGTCTCGCTTCGCTTCTGTGATTC-GACTTGTTCCGCCGTTGGATCTGGTGGAACGGAG
12_VM_5C        CGGTTCCTTCCCGTCATACTCATTGCCATTGGAGCTGAAAGGTCGAATTGCTGCTGCCTTGGGTCTCGCTTCGCTTCTGTGATTC-GACTTGTTCCGCCGTTGGATCTGGTGGAACGGAG
14_STDO_28      CGGTTCCTTCCCGTCATACTCATTGCCATTGGAGCTGAAAGGTCGAATTGCTGCTGCCTTGGGTCTCGCTTCGCTTCTGTGATTC-GACTTGTTCCGCCGTTGGATCTGGTGGAACGGAG
12_VM_5F        CGGTTCCTTCCCGTCATACTCATTGCCATTGGAGCTGAAAGGTCGAATTGCTGCTGCCTTGGGTCTCGCTTCGCTTCTGTGATTC-GACTTGTTCCGCCGTTGGATCTGGTGGAACGGAG
12_VM_5E        CGGTTCCTTCCCGTCATACTCATTGCCATTGGAGCTGAAAGGTCGAATTGCTGCTGCCTTGGGTCTCGCTTCGCTTCTGTGATTC-GACTTGTTCCGCCGTTGGATCTGGTGGAACGGAG
12_AGC_13C      CGGTTCCTTCCCGTCATACTCATTGCCATTGGAGCTGAAAGGTCGGATTGCTGCTGCCTTGGGTCTCGCTTCGCTTCTGTGATTCGGATTTGTTCCGCCGTTGGATCTGGTGGAACGGAG
12_VM_4F        CGGTTCCTTCCCGTCATACTCATTGCCATTGGAGCTGAAAGGTCGAATTGCTGCTGCCTTGGGTCTCGCTTCGCTTCTGTGATTCGGATTTGTTGCGCCGTTTGATCTGGTGGAACGGAG
ST_5548         CGGTTCCTTCCCGTCATACTCATTGCCATTGGAGCTGAAAGGTCGGATTGCTGCTGCCTTGGGTCTCGCTTCGCTTCTGTGATTCGGATTTGTTGCGCCGTTTGATCTCGTGGAACGGAG
14_AQ-1         CGGTTCCTTCCCGTCATACTCATTGCCATTGGAGCTGAAAGGTCGAATTGCTGCTGCCTTGGGTCTCGCTTCGCTTCTGTGATTC-GACTTGTTCCGCCGTTGGATCTGGTGGAACGGAG
14_STIRLO_13    CGGTTCCTTCCCGTCATACTCATTGCCATTGGAGCTGAAAGGTCGAATTGCTGCTGCCTTGGGTCTCGCTTCGCTTCTGTGATTC-GACTTGTTCCGCCGTTGGATCTGGTGGAACGGAG
15_PN_3         CGGTTCCTTCCCGTCATACTCATTGCCATTGGAGCTGAAAGGTCGAATTGCTGCTGCCTTGGGTCTCGCTTCGCTTCTGTGATTC-GACTTGTTCCGCCGTTGGATCTGGTGGAACGGAG
STDP_04915      CGGTTCCTTCCCGTCATACTCATTGCCATTGGAGCTGAAAGGTCGAATTGCTGCTGCCTTGGGTCTCGCTTCGCTTCTGTGATTC-GACTTGTTCCGCCGTTGGATCTGGTGGAACGGAG
15_OU_1D        CGGTTCCTTCCCGTCATACTCATTGCCATTGGAGCTGAAAGGTCGAATTGCTGCTGCCTTGGGTCTCGCTTCGCTTCTGTGATTCGGATTTGTTGCGCCGTTGGATCTCGTGGAACGGAG
15-PQ-8B        CGGTTCCTTCCCGTCATACTCATTGCCATTGGAGCTGAAAGGTCGAATTGCTGCTGCCTTGGGTCTCGCTTCGCTTCTGTGATTC-GACTTGTTCCGCCGTTGGATCTGGTGGAACGGAG
15_PQ-2B        CGGTTCCTTCCCGTCATACTCATTGCCATTGGAGCTGAAAGGTCGAATTGCTGCTGCCTTGGGTCTCGCTTCGCTTCTGTGATTC-GACTTGTTCCGCCGTTGGATCTGGTGGAACGGAG
09-ASA-3a       CGGTTCCTTCCCGTCATACTCATTGCCATTGGAGCTGAAAGGTCGGATTGCTGCTGCCTTGGGTCTCGCTTCGCTTCTGTGATTC-GACTTGTTCCGCCGTTGGATCTGGTGGAACGGAG
13-AHJ-8C       CGGTTCCTTCCCGTCATACTCATTGCCATTGGAGCTGAAAGGTCGGATTGCTGCTGCCTTGGGTCTCGCTTCGCTTCTGTGATTC-GACTTGTTCCGCCGTTGGATCTGGTGGAACGGAG
14-STDK         CGGTTCCTTCCCGTCATACTCATTGCCATTGGAGCTGAAAGGTCGGATTGCTGCTGCCTTGGGTCTCGCTTCGCTTCTGTGATTC-GACTTGTTCCGCCGTTGGATCTGGTGGAACGGAG
10-BNE-35       CGGTTCCTTCCCGTCATACTCATTGCCATTGGAGCTGAAAGGTCGGATTGCTGCTGCCTTGGGTCTCGCTTCGCTTCTGTGATTC-GACTTGTTCCGCCGTTGGATCTGGTGGAACGGAG
15-PQ-6A        CGGTTCCTTCCCGTCATACTCATTGCCATTGGAGCTGAAAGGTCGGATTGCTGCTGCCTTGGGTCTCGCTTCGCTTCTGTGATTC-GACTTGTTCCGCCGTTGGATCTGGTGGAACGGAG
09-ASA-10b.pz   CGGTTCCTTCCCGTCATACTCATTGCCATTGGAGCTGAAAGGTCGGATTGCTGCTGCCTTGGGTCTCGCTTCGCTTCTGTGATTC-GACTTGTTCCGCCGTTGGATCTGGTGGAACGGAG
12-VM-7A        CGGTTCCTTCCCGTCATACTCATTGCCATTGGAGCTGAAAGGTCGGATTGCTGCTGCCTTGGGTCTCGCTTCGCTTCTGTGATTC-GACTTGTTCCGCCGTTGGATCTGGTGGAACGGAG
14-EG-A1        CGGTTCCTTCCCGTCATACTCATTGCCATTGGAGCTGAAAGGTCGGATTGCTGCTGCCTTGGGTCTCGCTTCGCTTCTGTGATTC-GACTTGTTCCGCCGTTGGATCTGGTGGAACGGAG
09-CB1          CGGTTCCTTCCCGTCATACTCATTGCCATTGGAGCTGAAAGGTCGGATTGCTGCTGCCTTGGGTCTCGCTTCGCTTCTGTGATTC-GACTTGTTCCGCCGTTGGATCTGGTGGAACGGAG
14-AK1b         CGGTTCCTTCCCGTCATACTCATTGCCATTGGAGCTG-AAGGTCGGATTGCTGCTGCCTTGGGTTTCGCTTCGCTTCTGTGATTC-GATTTGTTCCACCGTTGGACCTGGTGGAACGGAG
consensus       *************************************.*******.******************.******************** **.*****.*.*****.**.**.***********
                ......1450......1460......1470......1480......1490......1500......1510......1520......1530......1540......1550......1560
```

```
gi|110265120|gb  A------TATTCGACCCCGGAATCCCCGAGTCCCGTCT-GCTCGA-------CAAACAGCCCGCCTTTGCA-------------------------------------------------
12-VM-4J         A------TATTCGACCCCGGAATCCCCGAGTCCCGTCT-GCTCGA-------CAAACAGCCCGCCTTTGCA-------------------------------------------------
12_VM_5A         A------TATTCGACCCCGGAATCCCCGAGTCCCGTCT-GCTCGA-------CAAACAGCCCGCCTTTGCA-------------------------------------------------
12_VM_5C         A------TATTCGACCCCGGAATCCCCGAGTCCCGTCT-GCTCGA-------CAAACAGCCCGCCTTTGCA-------------------------------------------------
14_STDO_28       A------TATTCGACCCCGGAATCCCCGAGTCCCGTCT-GCTCGA-------CAAACAGCCCGCCTTTGCA-------------------------------------------------
12_VM_5F         A------TATTCGACCCCGGAATCCCCGAGTCCCGTCT-GCTCGA-------CAAACAGCCCGCCTTTGCA-------------------------------------------------
12_VM_5E         A------TATTCGACCCCGGAATCCCCGAGTCCCGTCT-GCTCGA-------CAAACAGCCCGCCTTTGCA-------------------------------------------------
12_AGC_13C       A------TATTCGACCCCGGAATCCCCGAGTCCCGTCT-GCTCGA-------CAAACAGCCCGCCTTTGCA-------------------------------------------------
12_VM_4F         A------TATTCGACCCCGGAATCCCCGAGTCCCGTCT-GCTCGA-------CAAACAGCCCGCCTTTGCA-------------------------------------------------
ST_5548          A------TATTCGACCCCGGAATCCCCGAGTCCCGTCTGGCTCGA-------CAAACAGCCCGCCTTTGCA-------------------------------------------------
14_AQ-1          A------TATTCGACCCCGGAATCCCCGAGTCCCGTCT-GCTCGACAAACAGCAAACAGCCCGCCTTTGCA-------------------------------------------------
14_STIRLO_13     A------TATTCGACCCCGGAATCCCCGAGTCCCGTCT-GCTCGA-------CAAACAGCCCGCCTTTGCA-------------------------------------------------
15_PN_3          A------TATTCGACCCCGGAATCCCCGAGTCCCGTCT-GCTCGA-------CAAACAGCCCGCCTTTGCA-------------------------------------------------
STDP_04915       A------TATTCGACCCCGGAATCCCCGAGTCCCGTCT-GCTCGA-------CAAACAGCCCGCCTTTGCA-------------------------------------------------
15_OU_1D         A------TATTCGACCCCGGAAACCCCGAGTCCCGTCTGGCTCGA-------CAAACAGCCCGCCTTTGCA-------------------------------------------------
15-PQ-8B         ATAGTTCTATTCGACCCCGGAATCCCCGAGTCCCGTCT-GCTCGA-------CAAACAGCCCGCCTTTGCAGAGGATTCAGTTCGGCCGACCATTAGTCATCGCCAAGCCGACCAGTTGA
15_PQ-2B         ATAGTTCTATTCGACCCCGGAATCCCCGAGTCCCGTCT-GCTCGA-------CAAACAGCCCGCCTTTGCAGAGGATTCAGTTCGGCCGACCATTAGTCATCGCCAAGCCGACCAGTTGA
09-ASA-3a        A------TATTCGACCCCGGAATCCCCGAGTCCCGTCT-GCTCGA-------CAAACAGCCCGCCTTTGCA-------------------------------------------------
13-AHJ-8C        A------TATTCGACCCCGGAATCCCCGAGTCCCGTCT-GCTCGA-------CAAACAGCCCGCCTTTGCA-------------------------------------------------
14-STDK          A------TATTCGACCCCGGAATCCCCGAGTCCCGTCT-GCTCGA-------CAAACAGCCCGCCTTTGCA-------------------------------------------------
10-BNE-35        A------TATTCGACCCCGGAATCCCCGAGTCCCGTCT-GCTCGA-------CAAACAGCCCGCCTTTGCA-------------------------------------------------
15-PQ-6A         A------TATTCGACCCCGGAATCCCCGAGTCCCGTCT-GCTCGA-------CAAACAGCCCGCCTTTGCA-------------------------------------------------
09-ASA-10b.pz    A------TATTCGACCCCGGAATCCCCGAGTCCCGTCT-GCTCGA-------CAAACAGCCCGCCTTTGCA-------------------------------------------------
12-VM-7A         A------TATTCGACCCCGGAATCCCCGAGTCCCGTCT-GCTCGA-------CACTCAGCCCGCCTTTGCA-------------------------------------------------
14-EG-A1         A------TATTCGACCCCGGAATCCCCGAGTCCCGTCT-GCTCGAC------CCCACAGCCCGCCTTTGCA-------------------------------------------------
09-CB1           A------TATTCGACCCCGGAATCCCCGAGTCCCGTCT-GCTCGA-------CAAACAGCCCGCCTTTGCA-------------------------------------------------
14-AK1b          A------TATTCGACCCCGGAATCCCCGAGTCCCGTCTGGCTGGA-------CAAACAGCCCGCCTTTGCA-------------------------------------------------
consensus        *      ***************.*************** ***.**       *...***************
                 ......1570......1580......1590......1600......1610......1620......1630......1640......1650......1660......1670......1680

gi|110265120|gb  -----------------------------------------------------------------------------------------------------TACAAGTAAGGTATCACCT
12-VM-4J         -----------------------------------------------------------------------------------------------------TACAAGTACGGTATCACC-
12_VM_5A         -----------------------------------------------------------------------------------------------------TACAAGTACGGTATCACC-
12_VM_5C         -----------------------------------------------------------------------------------------------------TACAAGTACGGTATCACC-
14_STDO_28       -----------------------------------------------------------------------------------------------------TACAAGTACGGTATCACC-
12_VM_5F         -----------------------------------------------------------------------------------------------------TACAAGTACGGTATCACC-
12_VM_5E         -----------------------------------------------------------------------------------------------------TACAAGTACGGTATCACC-
12_AGC_13C       -----------------------------------------------------------------------------------------------------TACAA-----GTATCACC-
12_VM_4F         -----------------------------------------------------------------------------------------------------TACAAGTACGGTATCACC-
ST_5548          -----------------------------------------------------------------------------------------------------TACAAGTACGGTATCACC-
14_AQ-1          -----------------------------------------------------------------------------------------------------TACAAGTACGGTATCACCT
14_STIRLO_13     -----------------------------------------------------------------------------------------------------TACAAGTACGGTATCACC-
15_PN_3          -----------------------------------------------------------------------------------------------------TACAAGTAAGGTATCACCT
STDP_04915       -----------------------------------------------------------------------------------------------------TACAAGTACGGTATCACC-
15_OU_1D         -----------------------------------------------------------------------------------------------------TACAAGTACGGTATCACCT
15-PQ-8B         GTGCCAAGCCTACCACTCAAAAGTTCGGCACACCACTTGAATTCTTTCTCT---------------------------------------------------------------------
15_PQ-2B         GTGCCAAGCCTACCACTCAAAAGTTCGGCACACCACTTGAATTCTTTCTCTCTTTAACACCCCACACCAACATGTGAGTAACGACTATCCACGCCTTTGCATACAAGTACGGTATCACCT
09-ASA-3a        -----------------------------------------------------------------------------------------------------TACAA-----GTATCACCT
13-AHJ-8C        -----------------------------------------------------------------------------------------------------TACAA-----GTATCACCT
14-STDK          -----------------------------------------------------------------------------------------------------TACAA-----GTATCACCT
10-BNE-35        -----------------------------------------------------------------------------------------------------TACAA-----GTATCACCT
15-PQ-6A         -----------------------------------------------------------------------------------------------------TACAA-----GTATCACCT
09-ASA-10b.pz    -----------------------------------------------------------------------------------------------------TACAA-----GTATCACCT
12-VM-7A         -----------------------------------------------------------------------------------------------------TACAA-----GTATCACCT
14-EG-A1         -----------------------------------------------------------------------------------------------------TACAA-----GTATCACCT
09-CB1           -----------------------------------------------------------------------------------------------------TACAA-----GTATCACCT
14-AK1b          -----------------------------------------------------------------------------------------------------TACAAGTACGGTATCACCT
consensus                                                                                                             ...................
                 ......1690......1700......1710......1720......1730......1740......1750......1760......1770......1780......1790......1800
```

```
gi|110265120|gb    CACTTCTCAGGTTTGTAGCATTCACCACTTCGACCAGGTCAGGTCAGGCCCACCCCCGTCAACCCTCCAACAACCATACCTGGTAGTTCTACCCATCCACCGACTTCGTCATCGACGATA
12-VM-4J           ----TC---------AAGCATTCACCACTTCGACCAGGTCAGGTCAGGCCCACCCCCGTCAACCAT-CAACAACCATACCCGGTAGT---------------------------------
12_VM_5A           ----TC---------AAGCATTCACCACTTCGACCAGGTCAGGTCAGGCCCACCCCCGTCAACCAT-CAACAACCATACCCGGTAGTTCTACCCATCCCACGACTT--------------
12_VM_5C           ----TC---------AAGCATTCACCACTTCGACCAGGTCAGGTCAGGCCCACCCCCGTCAACCAT-CAACAACCATACCCGGTAGTTCTACCCATCCACCGACTTCGTCATCG------
14_STDO_28         ----TC---------AAGCATTCACCACTTCGACCAGGTCAGGTCAGGCCCACCCCCGTCAACCAT-CAACAACCATACCCGGTAGTTCTACCCATCCACCGACTTCGTCACGAC-----
12_VM_5F           ----TC---------AAGCATTCACCACTTCGACCAGGTCAGGTCAGGCCCACCCCCGTCAACCAT-CAACAACCATACCCGGTAGTTCTACCCATCCACCGACTTCGTCTCA-------
12_VM_5E           ----TC---------AAGCATTCACCACTTCGACCAGGTCAGGTCAGGCCCACCCCCGTCAACCAT-CAACAACCATACCCGGTAGTTCTACCCATCCCACGACTTCG------------
12_AGC_13C         ----TC---------AAGCATTCACCACTTCGACCAGGTCAGGTCAGGCCCACCCCCGTCAACCAT-CAACAACCATACCCGGTAGTTCTACCCATCCACCGACTTCGTCATCGAATTAC
12_VM_4F           ----TC---------AAGCATTCACCACTTCGACCAGGTCAGGTCAGGCCCACCCCCGTCAACCAT-CAACAACCATACCCGGTAGTTCTACCCATCCACCGACTTCGTCATCGACGATA
ST_5548            ----TC---------AAGCATTCACCACTTCGACCAGGTCAGGTCAGGCCCACCCCCGTCAACCAT-CAACAACCATACCCGGTAGTTCTACCCATCCACCGACTTCGTCATCGACA---
14_AQ-1            CACTTCTCAGGTTTGTAGCATTCACCACTTCGACCAGGTCAGGTCAGGCCCACCCCCGTCAACCCTCCAACAACCATACCTGGTAGTTCTACCCATCCACCGACTTCGTCATCGAC----
14_STIRLO_13       ----TC---------AAGCATTCACCACTTCGACCAGGTCAGGTCAGGCCCACCCCCGTCAACCAT-CAACAACCATACCCGGTAGTTCTACCCATCCACCGACTTCGTCATCGACATAC
15_PN_3            CTCATC---------AAGCATTCACCACTTCGACCAGGTCAGGTCAGGCCCACCCCCGTCAACCAT-CAACAACCATACCCGGTAGTTCTACCCATCCACCGACTTCGTCATCGA-----
STDP_04915         ----TC---------AAGCATTCACCACTTCGACCAGGTCAGGTCAGGCCCACCCCCGTCAACCAT-CAACAACCATACCCGGTAGTTCTACCCATCCACCGACTTCGTCATCGA-----
15_OU_1D           CACTTCTCAGGTTTGTAGCATTCACCACTTCGAC-----CAGGTCAGGCCCACCCCCGTCAACCCTCCAACAACCATACCTGGTAGTTCTACCCATCCACCGACTTCGTCATC-------
09-ASA-3a          C--------------AAGCATTCACCACTTCGACCAGGTCAGGTCAGGCCCACCCCCGTCAACCCTCCAACAACCATACCTGGTAGTTCTACCCATCCACCGCCTTCGTCATCGACCATA
13-AHJ-8C          C--------------AAGCATTCACCACTTCGACCAGGTCAGGTCAGGCCCACCCCCGTCAACCCTCCA---------------------------------------------------
14-STDK            C--------------AAGCATTCACCACTTCGACCAGGTCAGGTCAGGCCCACCCCCGTCAACCCTCCAACACC----------------------------------------------
10-BNE-35          C--------------AAGCATTCACCACTTCGACCAGGTCAGGTCAGGCCCACCCCCGTCAACCCTCCAACACC----------------------------------------------
15-PQ-6A           C--------------AAGCATTCACCACTTCGACCAGGTCAGGTCAGGCCCACCCCCGTCAACCCTCCAACAC-----------------------------------------------
09-ASA-10b.pz      C--------------AAGCATTCACCACTTCGACCAGGTCAGGTCAGGCCCACCCCCGTCAACCCTCCAACACCATACCTGTATC-----------------------------------
12-VM-7A           C--------------AAGCATTCACCACTTCGACCAGGTCAGGTCAGGCCCACCCCCGTC------------------------------------------------------------
14-EG-A1           C--------------AAGCATTCACCACTTCGACCAGGTCAGGTCAGGCCCACCCCCGTCAACCCTCCAGCAACCATA------------------------------------------
09-CB1             C--------------AAGCATTCACCACTTCGACCAGGTCAGGTCAGGCCCACCCCCGTCAACCCTCCAACAACCATACCTGGTAGTTCTACCCATCCACCGCCTTCGTCATCGACCATA
14-AK1b            CACTTCTCAGGTTTGTAGCATTCACCACTTCGA------CCGGTCAGGCCCACCCCCGTCAACCAT-CAA--------------------------------------------------
consensus          .   ..         ................................................... ............. .............................
                   ......1810......1820......1830......1840......1850......1860......1870......1880......1890......1900......1910......1920

gi|110265120|gb    CCGGCAGATCGCTCATACGAGCACGACTTCACCTCTCATCACCACCCCACACCGCCACCACAACCATGCGCTTCTTCAGCAAGAAGTCGCCGGCCGCGGTCGTCGACTCCACCGCACCCT
12-VM-4J           ------------------------------------------------------------------------------------------------------------------------
12_VM_5A           ------------------------------------------------------------------------------------------------------------------------
12_VM_5C           ------------------------------------------------------------------------------------------------------------------------
14_STDO_28         ------------------------------------------------------------------------------------------------------------------------
12_VM_5F           ------------------------------------------------------------------------------------------------------------------------
12_VM_5E           ------------------------------------------------------------------------------------------------------------------------
12_AGC_13C         CCGGCA------------------------------------------------------------------------------------------------------------------
12_VM_4F           CCGGCA------------------------------------------------------------------------------------------------------------------
ST_5548            ------------------------------------------------------------------------------------------------------------------------
14_AQ-1            ------------------------------------------------------------------------------------------------------------------------
14_STIRLO_13       CCCGGCA-----------------------------------------------------------------------------------------------------------------
15_PN_3            ------------------------------------------------------------------------------------------------------------------------
STDP_04915         ------------------------------------------------------------------------------------------------------------------------
15_OU_1D           ------------------------------------------------------------------------------------------------------------------------
15-PQ-8B           ------------------------------------------------------------------------------------------------------------------------
15_PQ-2B           ------------------------------------------------------------------------------------------------------------------------
09-ASA-3a          CCGGCAGATCGCTCATACGAACACGACTTCACCTCTCATCACCACCCCACACCGCCACCACAACCATGCGCTTCTTCAGCAAGAAGTCGCCGGCCGCGGTCGTCGACTCCACCGCACCCT
13-AHJ-8C          ------------------------------------------------------------------------------------------------------------------------
14-STDK            ------------------------------------------------------------------------------------------------------------------------
10-BNE-35          ------------------------------------------------------------------------------------------------------------------------
15-PQ-6A           ------------------------------------------------------------------------------------------------------------------------
09-ASA-10b.pz      ------------------------------------------------------------------------------------------------------------------------
12-VM-7A           ------------------------------------------------------------------------------------------------------------------------
14-EG-A1           ------------------------------------------------------------------------------------------------------------------------
09-CB1             CCGGCAGATCGCTCATACGAACACGACTTCACCTCTCATCACCACCCCACACCGCCACCACAACCATGCGCTTCTTCAGCAAGAAGTCGCCGGCCGCGGTCGTCGACTCCACCGCACCCT
14-AK1b            ------------------------------------------------------------------------------------------------------------------------
consensus
                   ......1930......1940......1950......1960......1970......1980......1990......2000......2010......2020......2030......2040
```
